# Phylogenetic placement and comparative analysis of the mitochondrial genomes of Idiostoloidea (Hemiptera: Heteroptera)

**DOI:** 10.1002/ece3.11328

**Published:** 2024-05-02

**Authors:** Danli Zhang, XiaoYan Chen, Jingjing Yang, Wenbo Yi, Qiang Xie, HuanHuan Yang, Merrill H. Sweet, Wenjun Bu, Teng Li

**Affiliations:** ^1^ College of Biological Sciences and Technology Taiyuan Normal University Jinzhong China; ^2^ Institute of Entomology, College of Life Sciences Nankai University Tianjin China; ^3^ School of Bioengineering Qilu University of Technology (Shandong Academy of Sciences) Jinan China; ^4^ Department of Entomology, Plant Pathology, and Weed Science New Mexico State University Las Cruces New Mexico USA; ^5^ School of Biological Sciences University of Auckland Auckland New Zealand

**Keywords:** divergence time, Idiostoloidea, mitogenome, phylogeny, ribosome RNA

## Abstract

The classification system and the higher level phylogenetic relationships of Pentatomomorpha, the second largest infraorder of Heteroptera (Insecta: Hemiptera), have been debated and remain controversial over decades. In particular, the placement and phylogenetic relationship of Idiostoloidea are not well resolved, which hampers a better understanding of the evolutionary history of Pentatomomorpha. In this study, for the first time, we reported the complete mitochondrial genome for two narrowly distributed families of Idiostoloidea (including Idiostolidae and Henicocoridae), respectively. The length of the mitochondrial genome of *Monteithocoris hirsutus* and *Henicocoris* sp. is 16,632 and 16,013 bp, respectively. The content of AT is ranging from 75.15% to 80.48%. The mitogenomic structure of Idiostoloidea is highly conservative and there are no gene arrangements. By using the Bayesian inference, maximum likelihood, and Bayesian site‐heterogeneous mixture model, we inferred the phylogenetic relationships within Pentatomomorpha and estimated their divergence times based on concatenated mitogenomes and nuclear ribosomal genes. Our results support the classification system of six superfamilies within Pentatomomorpha and confirm the monophyletic groups of each superfamily, with the following phylogenetic relationships: (Aradoidea + (Pentatomoidea + (Idiostoloidea + (Coreoidea + (Pyrrhocoroidea + Lygaeoidea))))). Furthermore, estimated divergence times revealed that most pentatomomorphan superfamilies and families diverged during the Late Jurassic to Early Cretaceous, which coincides with the explosive radiation of angiosperms.

## Graphical abstract

We reported the complete mitochondrial genome for two narrowly distributed families of Idiostoloidea (including Idiostolidae and Henicocoridae), respectively. Our results support the classification system of six superfamilies within Pentatomomorpha and confirm the monophyletic groups of each superfamily, with the following phylogenetic relationships: (Aradoidea + (Pentatomoidea + (Idiostoloidea + (Coreoidea + (Pyrrhocoroidea + Lygaeoidea))))).

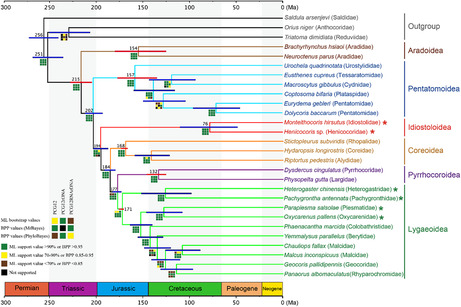

## 1 INTRODUCTION

Pentatomomorpha, the stink bugs and allies, is one of seven infraorders of Heteroptera (Insecta: Hemiptera) (Schuh & Slater, 1995). This group, mostly phytophagous, is the second largest heteropteran infraorder (Henry, 1997), with more than 16,000 species belonging to 42 families (Weirauch et al., 2019). Although the monophyly of Pentatomomorpha was well supported by both morphological characters and molecular data (Johnson et al., 2018; Li, Yang, et al., 2016; Schuh & Slater, 1995; Weirauch et al., 2019), the classification system and the higher level relationships within this group have been debated and remain contentious since Leston et al. (Leston et al., 1954) established this infraorder. Pentatomomorphan has been grouped into five (Schaefer, 1964, 1993; Schuh & Slater, 1995; Štys, 1961, 1967; Štys & Kerzhner, 1975), six (Carver et al., 1991; Cassis & Gross, 2002; Henry, 1997; Weirauch et al., 2019), or seven (Henry & Froeschner, 1988; Schuh, 1986) superfamilies (Table 1). Among these classification systems, the taxa of Aradoidea, Coreoidea, and Pentatomoidea are constant. Currently, six superfamilies (Aradoidea, Coreoidea, Idiostoloidea, Lygaeoidea, Pentatomoidea, and Pyrrhocoroidea) system proposed by Henry (1997) or five superfamilies (Lygaeoidea including Idiostoloidea) system proposed by Schaefer (1993) have been supported and accepted by nearly all subsequent authors (Weirauch et al., 2019; Weirauch & Schuh, 2011). Therefore, it is important to investigate the placement and phylogenetic relationship of Idiostoloidea within the Pentatomomorpha.

**TABLE 1:** Previous classification systems of Pentatomomorpha.

Five superfamilies		Six superfamilies		Seven superfamilies
Štys (1961, 1967) Štys and Kerzhner (1975)	Schaefer (1964, 1993) Schuh and Slater (1995)	Carver et al. (1991)	Henry (1997) Cassis and Gross (2002) Weirauch et al. (2019)	Schuh (1986)^a^ Henry and Froeschner (1988)
Aradoidea Pentatomoidea Idiostoloidea Coreoidea (including Lygaeoidea and Pyrrhocoroidea) Piesmatoidea	Aradoidea Pentatomoidea Coreoidea Pyrrhocoroidea Lygaeoidea (including Idiostoloidea and Piesmatoidea)	Aradoidea Pentatomoidea Idiostoloidea Coreoidea Lygaeoidea (including Pyrrhocoroidea) Piesmatoidea	Aradoidea Pentatomoidea Idiostoloidea Coreoidea Pyrrhocoroidea Lygaeoidea (including Piesmatoidea)	Aradoidea Pentatomoidea Idiostoloidea Coreoidea Pyrrhocoroidea Lygaeoidea Piesmatoidea

^a^ Schuh (1986) relegated Piesmatoidea to “*incertae sedis*.”

**FIGURE 1:**
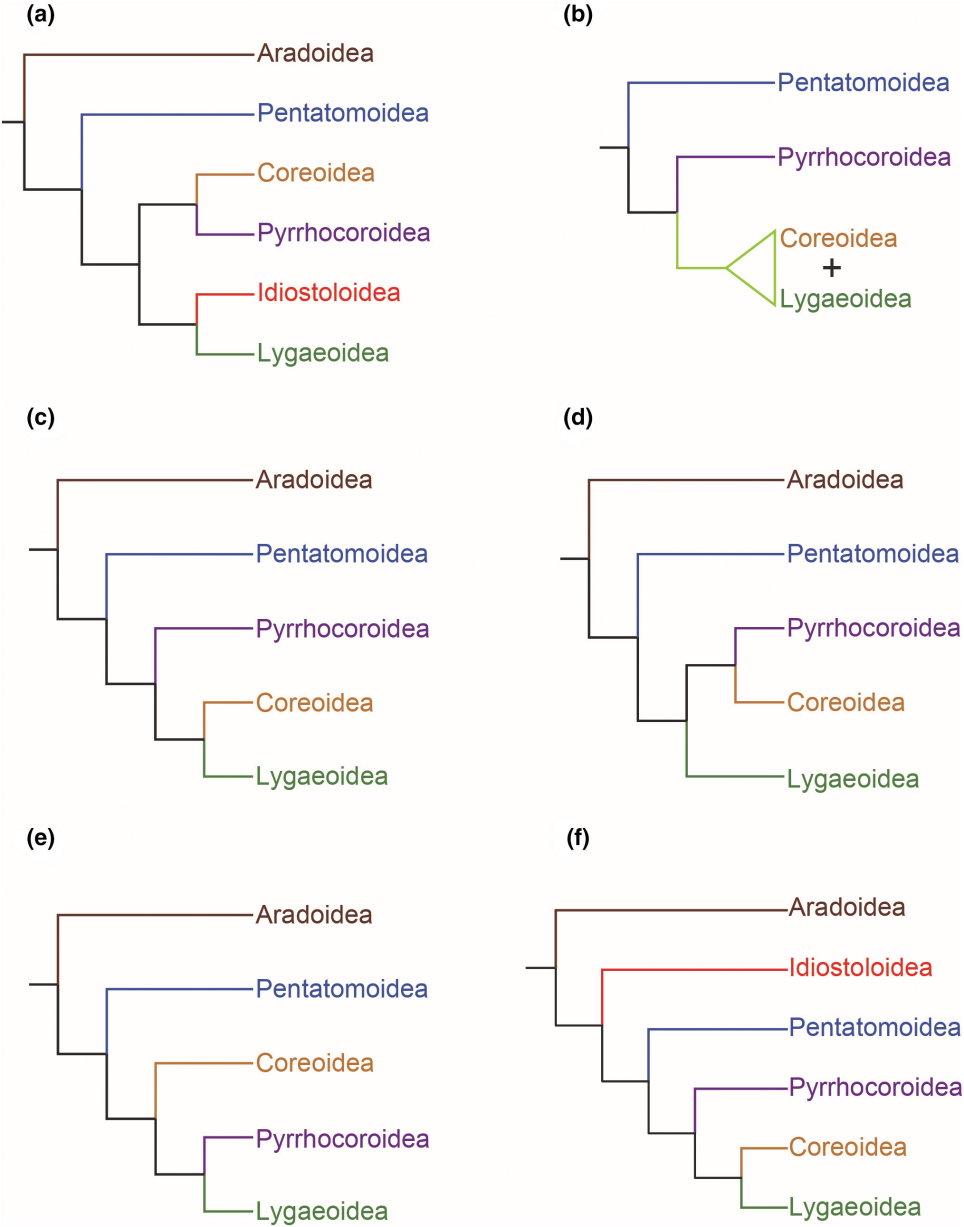
Proposed phylogenetic hypotheses about the superfamily relationships of Pentatomomorpha. (a) after Henry (1997); (b) after Xie et al. (2005); (c) after Hua et al. (2008); (d) after Tian et al. (2011); (e) after Wang et al. (2016); (f) after Weirauch et al. (2019).

Phylogenetic analyses of the superfamily or family level within Pentatomomorpha have been the subject of many studies based on molecular and morphological data, but relatively few studies have addressed the relationship of Idiostoloidea (Figure 1). The main reason for this may be due to the restricted distribution (southern South America and Australia) of Idiostoloidea and high difficulty in collection as the knowledge of biology of these organisms is limited (Cassis & Gross, 2002; Schuh & Slater, 1995). Henry (1997) proposed a sister group relationship between Idiostoloidea and Lygaeoidea, together with Coreoidea and Pyrrhocoroidea formed a monophyletic group based on morphological characters (Figure 1a). Weirauch et al. (2019) recently provided a comprehensive analysis of Heteroptera using molecular sequence data (partial sequence of 16S rDNA, 18S rDNA, and 28S rDNA) combined with morphological data treated Idiostoloidea as the sister group of the remaining Trichophora (including the superfamilies of Pentatomomorpha without Aradoidea) (Figure 1f), but the support value for this grouping is very low (bootstrap values lower than 50%), which indicated that these related nodes (Idiostoloidea, Pentatomoidea, and the rest of the Trichophora) are likely to be left unresolved, while increasing the number of characters may improve the support and resolution of phylogenetic inference (Delsuc et al., 2005; Wortley et al., 2005).

To date, the phylogenetic studies that have focused on resolving relationships across the infraorder, based on morphological characters (Henry, 1997) (Figure 1a), nuclear ribosomal gene of 18S rDNA (Xie et al., 2005) (Figure 1b), mitochondrial genomes (Hua et al., 2008) (Figure 1c), six Hox genes (Tian et al., 2011) (Figure 1d), and concatenated matrix (18S rRNA, 28S rRNA, and mitochondrial genes) (Wang et al., 2016) (Figure 1e), and combined morphological and molecular data analyses (Weirauch et al., 2019) (Figure 1f), recovered controversial relationships within Eutrichophora (Coreoidea, Lygaeoidea, and Pyrrhocoroidea), but consistently and strongly supported the Aradoidea as the sister group of Trichophora and the close relationship between Pentatomoidea and Eutrichophora. However, previous analyses using mitochondrial genome (mitogenome) sequences frequently reconstructed conflicting phylogenetic relationships within Eutrichophora, such as Hua et al. (2008) considered Coreoidea as sister group of Lygaeoidea, Yuan et al. (2015) suggested Coreoidea sister to Pyrrhocoroidea, and Liu et al. (2019) supported the sister group relationship between Pyrrhocoroidea and Lygaeoidea.

In the phylogenomic era, the mitogenome has become the most extensively used genomic resource in insects (Cameron, 2014), owing to its compact size (typically 15–18 kb), as well as their high copy number within the cell, which allows using the cost‐effective approach of shotgun assembly based on a relatively shallow next generation sequencing without any tedious PCR amplification (Kocher et al., 2014). However, mitogenome data alone have limited applicability in higher level phylogenetic inference of insects, because of these biases in accelerated substitution rates and compositional heterogeneity (Yang et al., 2018). On the other hand, nuclear ribosomal genes have been successfully used to study the phylogenetic relationships of various taxonomic levels (e.g., family level), due to their ubiquitous presence and relatively conservative function (Misof et al., 2007; Wu et al., 2016; Xie et al., 2009). Therefore, combining mitogenomes and nuclear ribosomal genes of 18S and 28S rDNA sequences can provide a more accurate picture of the evolutionary history of Pentatomomorpha.

## 2 MATERIALS AND METHODS

### 2.1 Taxon sampling

A total of 28 species of Heteroptera were sampled in this study, including 25 species from Pentatomomorpha as ingroups and three species from Leptopodomorpha and Cimicomorpha as outgroups (Table S1). The ingroups covered all six extant superfamilies of Pentatomomorpha. Among these, two mitogenomes and complete nuclear ribosomal sequences (18S rRNA, ITS1, 5.8S rRNA, ITS2, and 28S rRNA) of Idiostoloidea, and four mitogenomes of Lygaeoidea are reported here for the first time. All these newly sequenced species represent six different families, with the mitogenome of the rarely collected family of Henicocoridae being first reported here. The collecting information of six newly sequenced species is provided in Table S2, and specimens were preserved in 95% ethanol and stored at −20°C in the Insect Molecular Systematic Lab, Institute of Entomology, College of Life Sciences, Nankai University, Tianjin, China. Additionally, the mitogenomes of the other 22 heteropterans, coupled with the remaining 18S and 28S rDNA sequences (except for six species), were obtained from the National Center for Biotechnology Information (NCBI) database. The summary of sample information and corresponding GenBank accession numbers are listed in Table S1.

### 2.2 Sequencing and assembly

We extracted genomic DNA from thoracic muscle tissue using the DNeasy DNA Extraction kit (QIAGEN). Four mitogenomes of Lygaeoidea were generated by the amplification of four overlapping PCR fragments, and the primers used for amplification are listed in Table S3. Details of the PCR reaction conditions were described in our previous study (Li, Yang, et al., 2016) and then sequenced by ABI 3730XL capillary sequencer (Applied Biosystems). Raw sequence files were proofread and then assembled into contigs using BioEdit version 7.0.5.2 (Hall, 1999). Two entire mitogenomes of Idiostoloidea were obtained using Illumina HiSeq 2000 platform (Illumina, San Diego, CA) with 200 bp insert size and a paired‐end 100‐bp sequencing strategy at BGI‐Shenzhen, China. The sequence reads were first assessed with FastQC (Babraham Bioinformatics), adapter sequences and low‐quality reads were trimmed with Trimmomatic (Bolger et al., 2014), and then the remaining high‐quality reads were assembled using SOAPdenovo‐Trans (Xie et al., 2014). The resulting contigs were subsequently used as query in a blastn (Camacho et al., 2009) search against the reference mitogenomes and only those with a high hit score were retained as putative mitochondrial genomes. Additionally, the complete nuclear ribosomal gene sequences of Idiostoloidea were detected by using the assembled contigs BLAST against the 18S and 28S rDNAs of *Eurydema maracandica* (Yu et al., 2013).

### 2.3 Annotation and alignment

The assembled contigs and scaffolds were annotated for protein‐coding genes (PCGs) and rRNAs by BLAST searches of GenBank and alignment with homologous sequences, while tRNAs were identified according to tRNAscan‐SE version 1.21 (Lowe & Eddy, 1997). Each PCG was aligned separately based on amino acid translation using MAFFT (Katoh et al., 2002) as implemented in the TranslatorX online server (Abascal et al., 2010). Divergent and ambiguously aligned regions were removed from the protein alignment before back‐translating to nucleotides using Gblocks (Talavera & Castresana, 2007) within TranslatorX. The rRNA genes were individually aligned in MAFFT v7.0 online server (Katoh & Standley, 2013) using the G‐INS‐i algorithm. All aligned nucleotide sequences were checked manually in MEGA 7 (Kumar et al., 2016), and then concatenated to reconstruct the phylogeny excluding stop codons. The codon usage, nucleotide composition, and amino acid composition were calculated with MEGA 7 (Kumar et al., 2016). The formulas “AT skew = (A − T)/(A + T)” and “GC skew = (G − C)/(G + C)” were used to calculate strand asymmetry (Perna & Kocher, 1995).

### 2.4 Phylogenetic analyses

AliGROOVE (Kück et al., 2014) was employed to detect the heterogeneous sequence divergence within the alignment (e.g., each codon position of PCGs), while the third codon position of the PCGs showed strong sequence heterogeneity with mainly negative pairwise similarity scores (Figure S1). Thus, phylogenetic analyses were performed with three data matrices excluding the third codon and stop codon positions: (1) PCG12 matrix including the first and the second codon positions of the 13 PCGs; (2) PCG12rDNA matrix, including the PCG12 dataset with two rDNAs of 18S and 28S; (3) PCG12RNArDNA matrix, including the PCG12rDNA dataset with two rRNA genes of 16S and 12S. PartitionFinder 2 (Lanfear et al., 2017) was then used to detect the best partitioning schemes and corresponding nucleotide substitution models. The best‐fit partitioning schemes for these three datasets proposed by PartitionFinder 2 were utilized in the subsequent phylogenetic analyses (Table S4).

We used GPU MrBayes (Zhou et al., 2011) and PhyloBayes MPI version 1.7 (Lartillot et al., 2013) for Bayesian inference (BI), and raxmlGUI 1.2 (Silvestro & Michalak, 2012) for maximum likelihood (ML) analyses to reconstruct phylogenetic trees. MrBayes analysis was carried out with two simultaneous runs of 10,000,000 generations conducted for each matrix. Each set was sampled every 100 generations. In PhyloBayes analysis, we use the site‐heterogeneous model CAT + GTR, which has been suggested to reduce the artifacts resulting from mutational saturation and unequal patterns of substitution (Lartillot & Philippe, 2004; Song et al., 2016). Two independent chains were run in parallel until the analyses satisfactorily converged (maxdiff less than 0.3). For these two BI methods, trees that were sampled prior to stationarity (at 25% of the run) were discarded as burn‐in, and the remaining trees were used to construct a 50% majority‐rule consensus tree. For the ML analyses, GTR + I + Γ model was used for each matrix, and the node support was assessed with 1000 bootstrap replicates.

### 2.5 Molecular dating

We analyzed the concatenated DNA sequence alignment of PCG12RNArDNA using BEAST 1.8.0 under the uncorrelated lognormal relaxed clock model (Drummond & Rambaut, 2007). A Yule speciation process was used for the tree prior (Gernhard, 2008). The molecular clock was calibrated by six fossils with minimum age constraints, coupled with exponential priors on node times were used for these fossil calibrations (Table S5). Two replicate Markov chain Monte Carlo (MCMC) runs were performed with the tree and parameter values sampled every 1000 steps over a total of 50 million generations. Acceptable effective sample sizes (ESS) and convergence to the stationary distribution were checked by Tracer 1.7 (Rambaut et al., 2018). A maximum clade credibility tree was obtained from TreeAnnotator (Bouckaert et al., 2014) with a burn‐in of the first 10% of trees.

## 3 RESULTS AND DISCUSSION

### 3.1 Mitogenome comparisons

**FIGURE 2:**
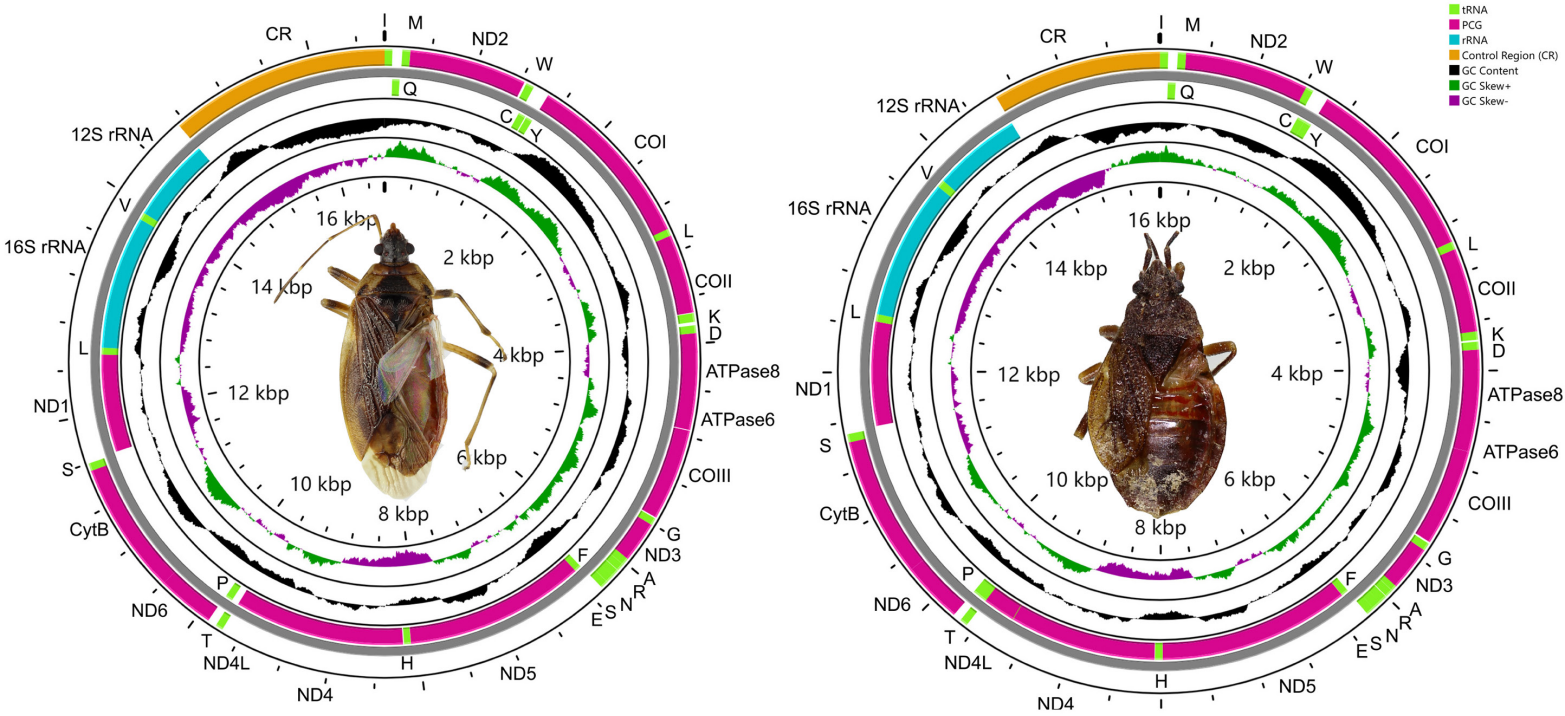
Structure of the mitochondrial genome of *Monteithocoris hirsutus* (left) and *Henicocoris* sp. (right). PCGs are shown as purple bars, rRNA genes as blue bars, tRNA genes as green bars, and control region as yellow bars. tRNAs are named using single‐letter amino acid abbreviations.

**FIGURE 3:**
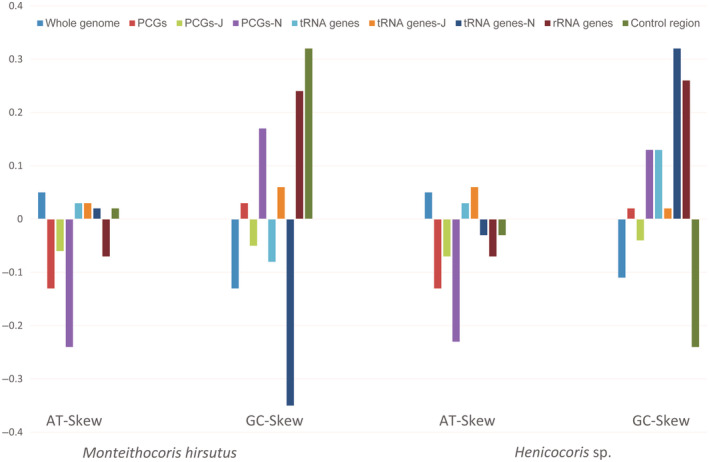
The AT skew and GC skew of *Monteithocoris hirsutus* (left) and *Henicocoris* sp. (right).

The complete mitochondrial genome of *Monteithocoris hirsutus* and *Henicocoris* sp. is circular double‐stranded molecules, which span 16,632 and 16,013 bp, respectively (Figure 2), matching very well with the ancestral gene arrangement of *Drosophila yakuba* (Clary & Wolstenholme, 1985). The nucleotide composition of *Monteithocoris hirsutus* and *Henicocoris* sp. is significantly AT biased ranging from 75.15% to 80.48% (Table 2), which is congruent with the rich A + T composition in insect mitogenomes (Cameron, 2014). The nucleotide skew statistics for the mitogenome of *Monteithocoris hirsutus* and *Henicocoris* sp. show that the whole genome, tRNA genes, and J‐strand tRNAs are AT skewed, whereas the other PCGs, J‐strand PCGs, N‐strand PCGs, and rRNAs are TA skewed. The difference in AT skew between *Monteithocoris hirsutus* and *Henicocoris* sp. is N‐strand tRNAs and control region. The N‐strand tRNAs and control region are AT skewed in *Monteithocoris hirsutus* and TA skewed in *Henicocoris* sp. (Figure 3). The GC skew of PCGs, N‐strand PCGs, J‐strand tRNAs, and rRNAs is positive, indicating a higher content of G nucleotides than C nucleotides, while GC skew of the whole genome and J‐strand PCGs are negative. The differences in GC skew between *Monteithocoris hirsutus* and *Henicocoris* sp. are observed in tRNAs, N‐strand tRNAs, and the control region. This kind of strand bias of nucleotide composition is likely related to asymmetric mutation processes during replication (Hassanin et al., 2005).

**TABLE 2:** Nucleotide composition and skewness of the *Monteithocoris hirsutus* and *Henicocoris* sp. mitochondrial genome.

Feature	Length(bp)	A%	C%	G%	T%	A + T%	AT skew	GC skew
*Monteithocoris hirsutus*								
Whole genome	16,632	40.48	12.83	9.79	36.90	77.38	0.05	−0.13
PCGs	11,052	33.14	11.59	12.27	43.00	76.14	−0.13	0.03
PCGs‐J	6810	35.29	13.07	11.78	39.87	75.15	−0.06	−0.05
PCGs‐N	4242	29.70	9.22	13.06	48.02	77.72	−0.24	0.17
tRNA genes	1460	40.62	11.23	9.52	38.63	79.25	0.03	−0.08
tRNA genes‐J	927	40.45	10.03	11.33	38.19	78.64	0.03	0.06
tRNA genes‐N	533	40.90	13.32	6.38	39.40	80.30	0.02	−0.35
rRNA genes	2062	36.57	8.15	13.39	41.90	78.47	−0.07	0.24
Control region	1893	41.10	6.66	12.94	39.30	80.40	0.02	0.32
*Henicocoris* sp.								
Whole genome	16,013	40.85	12.14	9.68	37.33	78.18	0.05	−0.11
PCGs	11,055	33.70	11.00	11.48	43.83	77.52	−0.13	0.02
PCGs‐J	6816	35.87	11.99	11.09	41.05	76.92	−0.07	−0.04
PCGs‐N	4239	30.20	9.14	12.10	48.29	78.49	−0.23	0.13
tRNA genes	1453	41.23	8.60	11.22	38.95	80.18	0.03	0.13
tRNA genes‐J	917	42.64	9.60	9.92	37.84	80.48	0.06	0.02
tRNA genes‐N	536	38.81	6.90	13.43	40.86	79.66	−0.03	0.32
rRNA genes	2054	36.37	7.98	13.58	42.06	78.43	−0.07	0.26
Control region	1374	38.86	12.45	7.57	41.12	79.99	−0.03	−0.24

**FIGURE 4:**
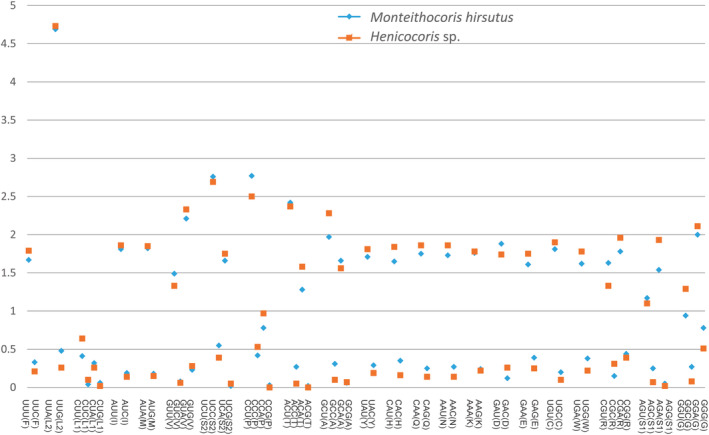
The RSCU in the *Monteithocoris hirsutus* and *Henicocoris* sp. mitochondrial PCGs.

**FIGURE 5:**
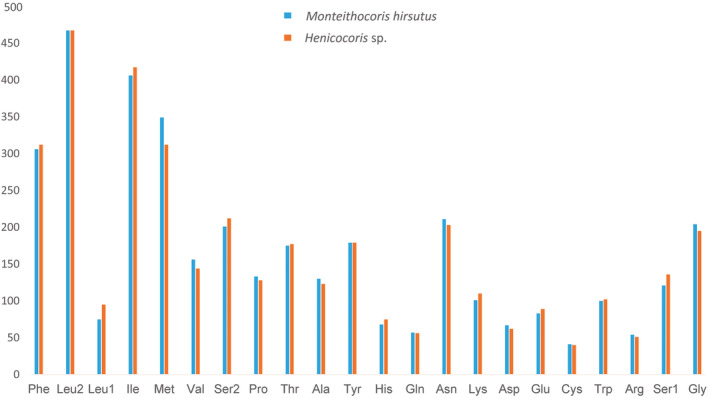
Amino acid composition in the *Monteithocoris hirsutus* and *Henicocoris* sp. mitogenomes. Codon families are provided on the *x*‐axis. Numbers of codons of each amino acid are provided on the *y*‐axis.

The mitogenome‐wide bias toward A + T is also reflected in the codon usage. We summarized the relative synonymous codon usage (RSCU) values for the mitogenomes of *Monteithocoris hirsutus* and *Henicocoris* sp. (Figure 4). According to the results of RSCU, synonymous codons ending with an A or U are more prevalent than those ending in a G or C, which were also observed in other heteropteran species (Li et al., 2013; Wang et al., 2016). For instance, UUU (RSCU = 1.67) of *Monteithocoris hirsutus* is more common than UUC (RSCU = 0.33) for Phe, which is the same with *Henicocoris* sp. (the RSCU of UUU is 1.79 while UUC is 0.21). Four most frequently used codons are AUA‐Met, AUU‐Ile, UUA‐Leu2, and UUU‐Phe (Figure 5), which are all composed wholly of A or U, which may also reflect the A + T bias of the whole mitogenomes.

The 13 PCGs of *Monteithocoris hirsutus* are 11,052 bp while the 13 PCGs of *Henicocoris* sp. are 11,055 bp, which is the typical order for Pentatomomorpha (Hua et al., 2008; Yuan et al., 2015; Zhao et al., 2018, 2019). All PCGs are initiated by ATN or TTG as a start codon. TAA has been assigned to the majority of the PCGs, while ND3 of *Monteithocoris hirsutus* and COI of *Henicocoris* sp. terminate with a single T residue (Tables S6 and S7). The incomplete termination codon could be presumed to be generated by the post‐transcriptional polyadenylation (Ojala et al., 1981). All the 22 tRNA genes are appeared in the mitogenome of *Monteithocoris hirsutus* and *Henicocoris* sp. (Figures S2 and S3), which could carry all 20 types of amino acids. The 22 tRNA genes, ranging from 61 to 74 bp, could be folded into almost perfect cloverleaf secondary structure with a few non‐Watson‐Crick matches (Figures S2 and S3) except tRNA‐Ser (GCU), which lacked the dihydrouridine (DHU) arm. This phenomenon found in tRNA‐Ser (GCU) has been widely reported in many other hemipterans (Zhang et al., 2018, 2019). The rRNA genes of 16S are assumed to be between tRNA‐Leu (UAG) and tRNA‐Val, while 12S rRNA genes are located at tRNA‐Val and control region (Figure 2). The rRNA genes of *Monteithocoris hirsutus* and *Henicocoris* sp. are 2062 and 2054 bp long, with the A + T percent of 78.47% and 78.43%, respectively, indicating a moderate AT preference.

### 3.2 Phylogenetic analyses

**FIGURE 6:**
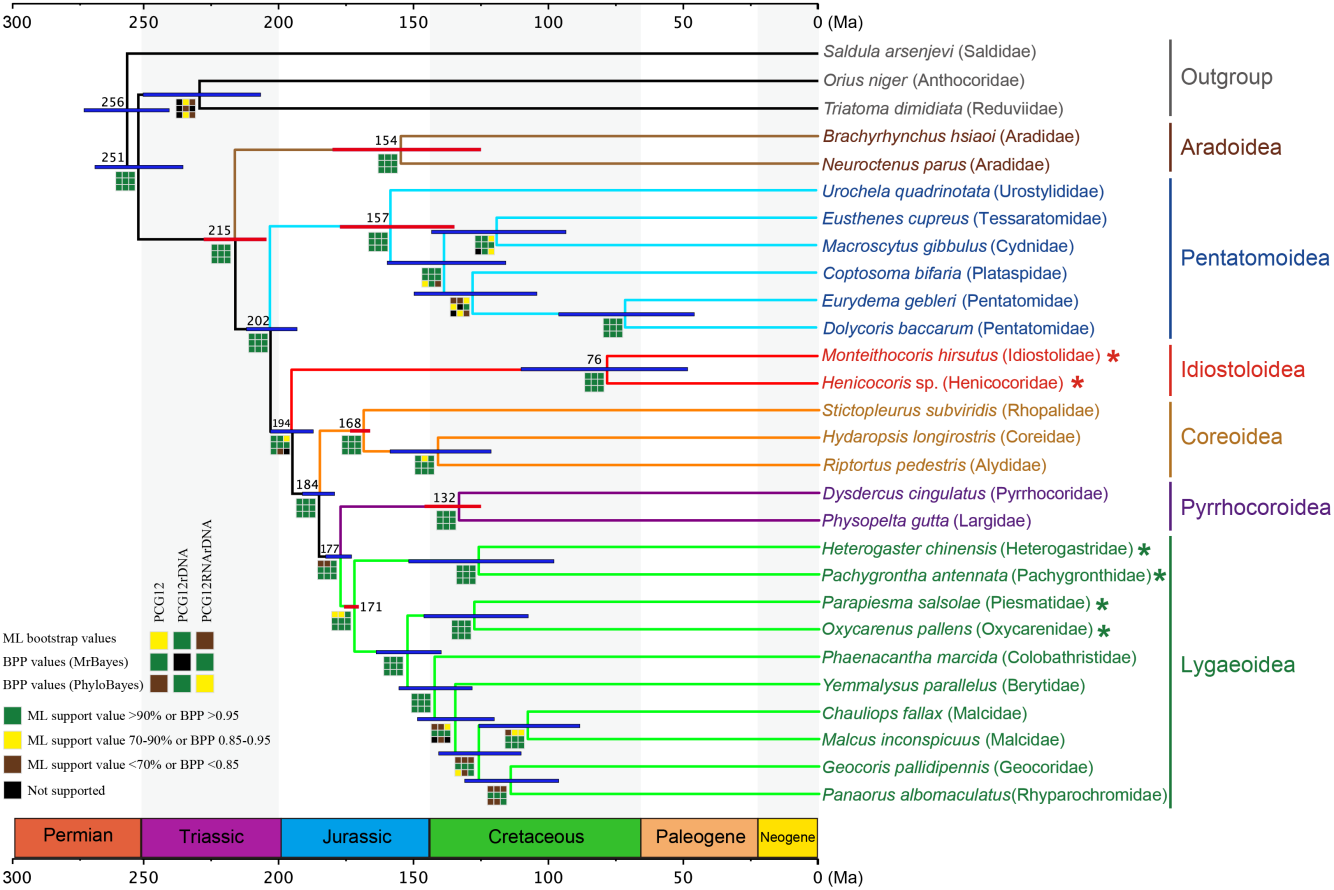
Phylogenetic chronogram of Pentatomomorpha based on mitochondrial genomes and nrDNAs (PCG12RNArDNA) by using BEAST. Red bars denote the calibration points, and blue bars indicate 95% mean confidence intervals. Colored cubes at the nodes indicate the support values (from top to bottom rows: ML support values, Bayesian posterior probabilities (BPP) of MrBayes, BPP of PhyloBayes) for different data matrices (from left to right columns: PCG12, PCG12rDNA, PCG12RNArDNA), respectively. Asterisks indicate new mitochondrial genomes sequenced in this study. The geological time scale is shown at the bottom.

Insect mitogenomes, especially within Hemiptera, are frequently characterized by accelerated substitution rates and compositional heterogeneity (e.g., A + T content heterogeneity), which can potentially return artifactual relationships in the higher level phylogenetic analyses (Bernt et al., 2013; Li et al., 2015; Yang et al., 2018). Bayesian analyses using site‐heterogeneous models implemented in PhyloBayes (e.g., CAT + GTR) can lessen the impact of compositional biases and alleviate related phylogenetic artifacts (e.g., long branch attraction) (Lartillot et al., 2007; Song et al., 2016; Timmermans et al., 2015). Therefore, we employ the homogeneous model (GTR + I + Γ) in both ML and MrBayes, as well as the heterogeneous model (CAT + GTR) in PhyloBayes to analyze the data matrices of PCG12, PCG12rDNA, and PCG12RNArDNA, with the results summarized in Figure 6. All nine phylogenetic trees revealed the same topology regarding superfamily‐level relationships except the position of Idiostoloidea in PCG12RNArDNA of PhyloBayes under the CAT + GTR model. The monophyly of Pentatomomorpha was strongly supported, and all six superfamilies (Aradoidea, Coreoidea, Idiostoloidea, Lygaeoidea, Pentatomoidea, and Pyrrhocoroidea) were recovered as monophyletic groups in our analyses. Aradoidea was supported as the sister group to the remaining lineages, which was consistent with previous studies (Henry, 1997; Hua et al., 2008; Tian et al., 2011; Wang et al., 2016; Weirauch et al., 2019; Ye et al., 2022). The superfamilies of Trichophora were retrieved as (Pentatomoidea + (Idiostoloidea + (Coreoidea + (Pyrrhocoroidea + Lygaeoidea)))), but Idiostoloidea was not supported as the sister group of Eutrichophora in PCG12RNArDNA of PhyloBayes under the CAT + GTR model. Our results of the placement of Idiostoloidea were congruent with the results of Ye et al. (2022), which were based on nuclear and mitochondrial PCGs and rRNA genes, but the mitogenome data of Idiostoloidea are limited in their study (i.e., they only have two partial PCGs represent for Henicocoridae).

The relationships among the three superfamilies Coreoidea, Lygaeoidea, and Pyrrhocoroidea within Eutrichophora remain highly debated and contentious (Henry, 1997; Hua et al., 2008; Liu et al., 2019; Tian et al., 2011; Weirauch et al., 2019; Yang et al., 2018; Zhao et al., 2021). All of our analyses recovered Coreoidea formed the sister group to the clade of Pyrrhocoroidea + Lygaeoidea, which received strong support in Bayesian analyses using both homogeneous and heterogeneous models, but weak support in ML bootstrap analyses (Figure 6). This relationship was also supported by previous analyses using mitogenomes with Bayesian inference under site‐heterogeneous models (Liu et al., 2019), mitochondrial PCGs and nuclear ribosomal genes (Wang et al., 2016), and 21 nuclear and mitochondrial genes (Li, Wang, et al., 2016). Within Lygaeoidea, our results highly supported a close relationship between Heterogastridae and Pachygronthidae, which together were placed at the basal lineage as the sister group to the remaining families of Lygaeoidea (Figure 6). Moreover, Piesmatidae was strongly supported as the sister group of Oxycarenidae, thereby confirming that Piesmatidae is a member of Lygaeoidea rather than being raised to a superfamily, as proposed by Štys (1961), Štys (1967), Štys and Kerzhner (1975), Carver et al. (1991), and Henry and Froeschner (1988). We also recovered the groups (Colobathristidae + (Berytidae + (Malcidae + (Geocoridae + Rhyparochromidae)))), but without strong support from either ML or PhyloBayes under the CAT + GTR model. However, our principal goal in this study was to investigate the phylogenetic position of Idiostoloidea and the evolutionary history of Pentatomomorpha. As we did not sample all the families of Eutrichophora and Lygaeoidea, a more thorough sampling of taxa is required to adequately resolve the higher level relationships within Eutrichophora and Lygaeoidea, respectively.

### 3.3 Divergence time estimation

The phylogenetic chronogram of Pentatomomorpha diversification based on PCG12RNArDNA matrix is shown in Figure 6. Our analyses suggested that the divergence of the infraorder Pentatomomorpha occurred 215 Ma (95% confidence interval (CI): 204–227 Ma) in the Late Triassic, which is consistent with recent studies based on multilocus (Li et al., 2017; Wang et al., 2016) and transcriptome data (Wang et al., 2019). The most recent common ancestor of Trichophora was estimated to have arisen 202 Ma (95% CI: 193–211 Ma). The divergence of the lineages leading to Idiostoloidea and Eutrichophora occurred 194 Ma (95% CI: 187–203 Ma) in the Early Jurassic. Within Eutrichophora, Coreoidea diverged from Lygaeoidea + Pyrrhocoroidea 184 Ma (95% CI: 179–191 Ma), followed by the split between Lygaeoidea and Pyrrhocoroidea 177 Ma (95% CI: 172–182 Ma) in the Middle Jurassic (Figure 6), which is close to the estimation of Liu et al. (2019) based on mitogenomes.

Our results indicated that the divergence of most pentatomomorphan superfamilies and families occurred from Late Jurassic to Early Cretaceous, which is coincident with the estimation of the angiosperm radiations (Li et al., 2019). The explosive radiation of angiosperms would offer a high diversity of heterogeneous niches and substantial nutrition resources to drive the evolution and diversification of Pentatomomorpha. Additionally, the split between Idiostolidae and Henicocoridae was estimated to have occurred 76 Ma (95% CI: 48–110 Ma) in Late Cretaceous, indicating that the divergence of Idiostoloidea is more recent than that of other superfamilies within Pentatomomorpha (132–171 Ma).

## 4 CONCLUSIONS

In this study, two mitogenomes and complete nuclear ribosomal sequences of Idiostoloidea and four mitogenomes of Lygaeoidea are reported here for the first time. Coupled with published data, phylogenetic analyses and divergence time were conducted in Pentatomomorpha. Our results confirm the monophyletic groups of each superfamily, with the following phylogenetic relationships: (Aradoidea + (Pentatomoidea + (Idiostoloidea + (Coreoidea + (Pyrrhocoroidea + Lygaeoidea))))). Furthermore, estimated divergence times revealed that most pentatomomorphan superfamilies and families diverged during the Late Jurassic to Early Cretaceous, which coincides with the explosive radiation of angiosperms. In comparison of mitogenomes, relatively little variation was observed in the length of PCGs, tRNAs, and rRNAs. The gene order matches very well with the ancestral gene arrangement of *Drosophila yakuba*. The AT content was significantly higher than the GC content. Our findings reveal the relationships among superfamilies, and more mitogenomes and nuclear genes should be sequenced to comprehensively understand the mitogenomic evolution and phylogenetic relationships of Pentatomomorpha.

## AUTHOR CONTRIBUTIONS

**Danli Zhang:** Data curation (equal); formal analysis (equal); investigation (equal); visualization (equal); writing – original draft (lead). **XiaoYan Chen:** Data curation (equal); investigation (equal). **Jingjing Yang:** Methodology (equal); software (equal). **Wenbo Yi:** Data curation (equal); methodology (equal). **Qiang Xie:** Methodology (equal). **HuanHuan Yang:** Formal analysis (equal). **Merrill H. Sweet:** Resources (equal). **Wenjun Bu:** Resources (equal); supervision (equal); writing – review and editing (equal). **Teng Li:** Resources (equal); supervision (equal); writing – review and editing (equal).

## CONFLICT OF INTEREST STATEMENT

The authors declare that they have no conflicting interests.

## Supporting information


Appendix S1.

## Data Availability

All the newly sequenced data have been submitted to GenBank. The accession numbers of nuclear ribosomal genes of *Monteithocoris hirsutus* and *Henicocoris* sp. are OR223290 and OR223291. The accession numbers of mitochondrial genome of *Monteithocoris hirsutus*, *Henicocoris* sp., *Heterogaster chinensis*, *Oxycarenus pallens*, *Pachygrontha antennata*, and *Parapiesma salsolae* are OR134606, OR189406, OR134605, OR134607, OR134608, and OR134773.

## References

[ece311328-bib-0001] Abascal, F. , Zardoya, R. , & Telford, M. J. (2010). TranslatorX: Multiple alignment of nucleotide sequences guided by amino acid translations. Nucleic Acids Research, 38(2), W7–W13. 10.1093/nar/gkq291 20435676 PMC2896173

[ece311328-bib-0002] Bernt, M. , Bleidorn, C. , Braband, A. , Dambach, J. , Donath, A. , Fritzsch, G. , Golombek, A. , Hadrys, H. , Juhling, F. , Meusemann, K. , Middendorf, M. , Misof, B. , Perseke, M. , Podsiadlowski, L. , von Reumont, B. , Schierwater, B. , Schlegel, M. , Schrodl, M. , Simon, S. , … Struck, T. H. (2013). A comprehensive analysis of bilaterian mitochondrial genomes and phylogeny. Molecular Phylogenetics and Evolution, 69(2), 352–364. 10.1016/j.ympev.2013.05.002 23684911

[ece311328-bib-0003] Bolger, A. M. , Lohse, M. , & Usadel, B. (2014). Trimmomatic: A flexible trimmer for Illumina sequence data. Bioinformatics, 30(15), 2114–2120. 10.1093/bioinformatics/btu170 24695404 PMC4103590

[ece311328-bib-0004] Bouckaert, R. , Heled, J. , Kuhnert, D. , Vaughan, T. , Wu, C. H. , Xie, D. , Suchard, M. A. , Rambaut, A. , & Drummond, A. J. (2014). BEAST 2: A software platform for Bayesian evolutionary analysis. PLoS Computational Biology, 10(4), e1003537. 10.1371/journal.pcbi.1003537 24722319 PMC3985171

[ece311328-bib-0005] Camacho, C. , Coulouris, G. , Avagyan, V. , Ma, N. , Papadopoulos, J. , Bealer, K. , & Madden, T. L. (2009). BLAST+: Architecture and applications. BMC Bioinformatics, 10(1), 421. 10.1186/1471-2105-10-421 20003500 PMC2803857

[ece311328-bib-0006] Cameron, S. L. (2014). Insect mitochondrial genomics: Implications for evolution and phylogeny. Annual Review of Entomology, 59(1), 95–117. 10.1146/annurev-ento-011613-162007 24160435

[ece311328-bib-0007] Carver, M. , Gross, G. , & Woodward, T. (1991). Hemiptera (Bugs, leafhoppers, cicadas, aphids, scale insects etc.). The insects of Australia: A textbook for students and research workers. Volume 1 (2nd ed., Ch.30, pp. 429–509, illus). Melbourne Univ. Press.

[ece311328-bib-0008] Cassis, G. , & Gross, G. (2002). Hemiptera: Heteroptera (Pentatomomorpha). CSIRO Publishing.

[ece311328-bib-0009] Clary, D. O. , & Wolstenholme, D. R. (1985). The mitochondrial DNA molecule of *drosophila yakuba*: Nucleotide sequence, Gene Organization, and genetic code. Journal of Molecular Evolution, 22(3), 252–271. 10.1007/BF02099755 3001325

[ece311328-bib-0010] Delsuc, F. , Brinkmann, H. , & Philippe, H. (2005). Phylogenomics and the reconstruction of the tree of life. Nature Reviews Genetics, 6(5), 361–375. 10.1038/nrg1603 15861208

[ece311328-bib-0011] Drummond, A. J. , & Rambaut, A. (2007). BEAST: Bayesian evolutionary analysis by sampling trees. BMC Ecology and Evolution, 7(1), 214. 10.1186/1471-2148-7-214 PMC2247476 17996036

[ece311328-bib-0012] Gernhard, T. (2008). The conditioned reconstructed process. Journal of Theoretical Biology, 253, 769–778. 10.1016/j.jtbi.2008.04.005 18538793

[ece311328-bib-0013] Hall, T. A. (1999). BioEdit: A user‐friendly biological sequence alignment editor and analysis program for windows 95/98/NT. Nucleic Acids Symposium Series, 41, 95–98. https://www.academia.edu/2034992/BioEdit_a_user_friendly_biological_sequence_alignment_editor_and_analysis_program_for_Windows_95_98_NT

[ece311328-bib-0014] Hassanin, A. , Leger, N. , & Deutsch, J. (2005). Evidence for multiple reversals of asymmetric mutational constraints during the evolution of the mitochondrial genome of Metazoa, and consequences for phylogenetic inferences. Systematic Biology, 54(2), 277–298. 10.1080/10635150590947843 16021696

[ece311328-bib-0015] Henry, T. J. (1997). Phylogenetic analysis of family groups within the infraorder Pentatomomorpha (Hemiptera: Heteroptera), with emphasis on the Lygaeoidea. Annals of the Entomological Society of America, 90(3), 275–301. 10.1093/aesa/90.3.275

[ece311328-bib-0016] Henry, T. J. , & Froeschner, R. C. (1988). Catalog of the Heteroptera, or true bugs, of Canada and the continental United States. Brill, Leiden, The Netherlands. Annals of the Entomological Society of America, 84(1), 132–134. https://www.biodiversitylibrary.org/part/180496

[ece311328-bib-0017] Hua, J. M. , Li, M. , Dong, P. Z. , Cui, Y. , Xie, Q. , & Bu, W. J. (2008). Comparative and phylogenomic studies on the mitochondrial genomes of Pentatomomorpha (Insecta: Hemiptera: Heteroptera). BMC Genomics, 9(1), 610. 10.1186/1471-2164-9-610 19091056 PMC2651891

[ece311328-bib-0018] Johnson, K. P. , Dietrich, C. H. , Friedrich, F. , Beutel, R. G. , Wipfler, B. , Peters, R. S. , Allen, J. M. , Petersen, M. , Donath, A. , Walden, K. K. O. , Kozlov, A. M. , Podsiadlowski, L. , Mayer, C. , Meusemann, K. , Vasilikopoulos, A. , Waterhouse, R. M. , Cameron, S. L. , Weirauch, C. , Swanson, D. R. , … Yoshizawa, K. (2018). Phylogenomics and the evolution of hemipteroid insects. Proceedings of the National Academy of Sciences, 115(50), 12775–12780. 10.1073/pnas.1815820115 PMC6294958 30478043

[ece311328-bib-0019] Katoh, K. , Misawa, K. , Kuma, K. , & Miyata, T. (2002). MAFFT: A novel method for rapid multiple sequence alignment based on fast Fourier transform. Nucleic Acids Research, 30(14), 3059–3066. 10.1093/nar/gkf436 12136088 PMC135756

[ece311328-bib-0020] Katoh, K. , & Standley, D. M. (2013). MAFFT multiple sequence alignment software version 7: Improvements in performance and usability. Molecular Biology and Evolution, 30(4), 772–780. 10.1093/molbev/mst010 23329690 PMC3603318

[ece311328-bib-0021] Kocher, A. , Kamilari, M. , Lhuillier, E. , Coissac, E. , Peneau, J. , Chave, J. , & Murienne, J. (2014). Shotgun assembly of the assassin bug Brontostoma colossus mitochondrial genome (Heteroptera, Reduviidae). Gene, 552(1), 184–194. 10.1016/j.gene.2014.09.033 25240790

[ece311328-bib-0022] Kück, P. , Meid, S. A. , Groß, C. , Wägele, J. W. , & Misof, B. (2014). AliGROOVE–visualization of heterogeneous sequence divergence within multiple sequence alignments and detection of inflated branch support. BMC Bioinformatics, 15(1), 294. 10.1186/1471-2105-15-294 25176556 PMC4167143

[ece311328-bib-0023] Kumar, S. , Stecher, G. , & Tamura, K. (2016). MEGA7: Molecular evolutionary genetics analysis version 7.0 for bigger datasets. Molecular Biology and Evolution, 33(7), 1870–1874. 10.1093/molbev/msw054 27004904 PMC8210823

[ece311328-bib-0024] Lanfear, R. , Frandsen, P. B. , Wright, A. M. , Senfeld, T. , & Calcott, B. (2017). PartitionFinder 2: New methods for selecting partitioned models of evolution for molecular and morphological phylogenetic analyses. Molecular Biology and Evolution, 34(3), 772–773. 10.1093/molbev/msw260 28013191

[ece311328-bib-0025] Lartillot, N. , Brinkmann, H. , & Philippe, H. (2007). Suppression of long‐branch attraction artefacts in the animal phylogeny using a site‐heterogeneous model. BMC Evolutionary Biology, 7(Suppl 1), S4. 10.1186/1471-2148-7-S1-S4 PMC1796613 17288577

[ece311328-bib-0026] Lartillot, N. , & Philippe, H. (2004). A Bayesian mixture model for across‐site heterogeneities in the amino‐acid replacement process. Molecular Biology and Evolution, 21(6), 1095–1109. 10.1093/molbev/msh112 15014145

[ece311328-bib-0027] Lartillot, N. , Rodrigue, N. , Stubbs, D. , & Richer, J. (2013). PhyloBayes MPI: Phylogenetic reconstruction with infinite mixtures of profiles in a parallel environment. Systematic Biology, 62(4), 611–615. 10.1093/sysbio/syt022 23564032

[ece311328-bib-0028] Leston, D. , Pendergrast, J. G. , & Southwood, T. R. E. (1954). Classification of the terrestrial Heteroptera (Geocorisae). Nature, 174(4419), 91–92. 10.1038/174091b0

[ece311328-bib-0029] Li, H. , Leavengood, J. M., Jr. , Chapman, E. G. , Burkhardt, D. , Song, F. , Jiang, P. , Liu, J. , Zhou, X. , & Cai, W. (2017). Mitochondrial phylogenomics of Hemiptera reveals adaptive innovations driving the diversification of true bugs. Proceedings of the Royal Society of London B: Biological Sciences, 284, 20171223. 10.1098/rspb.2017.1223 PMC5597834 28878063

[ece311328-bib-0030] Li, H. , Shao, R. , Song, N. , Song, F. , Jiang, P. , Li, Z. , & Cai, W. (2015). Higher‐level phylogeny of paraneopteran insects inferred from mitochondrial genome sequences. Scientific Reports, 5, 8527. 10.1038/srep08527 25704094 PMC4336943

[ece311328-bib-0031] Li, H. T. , Yi, T. S. , Gao, L. M. , Ma, P. F. , Zhang, T. , Yang, J. B. , Gitzendanner, M. A. , Fritsch, P. W. , Cai, J. , Luo, Y. , Wang, H. , van der Bank, M. , Zhang, S. D. , Wang, Q. F. , Wang, J. , Zhang, Z. R. , Fu, C. N. , Yang, J. , Hollingsworth, P. M. , … Li, D. Z. (2019). Origin of angiosperms and the puzzle of the Jurassic gap. Nature Plants, 5(5), 461–470. 10.1038/s41477-019-0421-0 31061536

[ece311328-bib-0032] Li, M. , Wang, Y. , Xie, Q. , Tian, X. , Li, T. , Zhang, H. , & Bu, W. (2016). Reanalysis of the phylogenetic relationships of the Pentatomomorpha (Hemiptera: Heteroptera) based on ribosomal, Hox and mitochondrial genes. Entomotaxonomia, 38(2), 81–91. 10.11680/entomotax.2016021

[ece311328-bib-0033] Li, T. , Gao, C. Q. , Cui, Y. , Xie, Q. , & Bu, W. J. (2013). The complete mitochondrial genome of the stalk‐eyed bug *Chauliops fallax* Scott, and the monophyly of Malcidae (Hemiptera: Heteroptera). PLoS One, 8(2), e55381. 10.1371/journal.pone.0055381 23390534 PMC3563593

[ece311328-bib-0034] Li, T. , Yang, J. , Li, Y. , Cui, Y. , Xie, Q. , Bu, W. , & Hillis, D. M. (2016). A mitochondrial genome of Rhyparochromidae (Hemiptera: Heteroptera) and a comparative analysis of related mitochondrial genomes. Scientific Reports, 6, 35175. 10.1038/srep35175 27756915 PMC5069475

[ece311328-bib-0035] Liu, Y. Q. , Li, H. , Song, F. , Zhao, Y. S. , Wilson, J. J. , & Cai, W. Z. (2019). Higher‐level phylogeny and evolutionary history of Pentatomomorpha (Hemiptera: Heteroptera) inferred from mitochondrial genome sequences. Systematic Entomology, 44(4), 810–819. 10.1111/syen.12357

[ece311328-bib-0036] Lowe, T. M. , & Eddy, S. R. (1997). tRNAscan‐SE: A program for improved detection of transfer RNA genes in genomic sequence. Nucleic Acids Research, 25(5), 955–964. 10.1093/nar/25.5.955 9023104 PMC146525

[ece311328-bib-0037] Misof, B. , Niehuis, O. , Bischoff, I. , Rickert, A. , & Staniczek, A. (2007). Towards an 18s phylogeny of hexapods: Accounting for group‐specific character covariance in optimized mixed nucleotide/doublet models. Zoology, 110(5), 409–429. 10.1016/j.zool.2007.08.003 17964130

[ece311328-bib-0038] Ojala, D. , Montoya, J. , & Attardi, G. (1981). tRNA punctuation model of RNA processing in human mitochondria. Nature, 290(5806), 470–474. 10.1038/290470a0 7219536

[ece311328-bib-0039] Perna, N. T. , & Kocher, T. D. (1995). Patterns of nucleotide composition at fourfold degenerate sites of animal mitochondrial genomes. Journal of Molecular Evolution, 41(3), 353–358. https://www.usualwant.com/article/10.1007/BF00186547 7563121 10.1007/BF00186547

[ece311328-bib-0040] Rambaut, A. , Drummond, A. J. , Xie, D. , Baele, G. , & Suchard, M. A. (2018). Posterior summarization in Bayesian phylogenetics using tracer 1.7. Systematic Biology, 67(5), 901–904. 10.1093/sysbio/syy032 29718447 PMC6101584

[ece311328-bib-0042] Schaefer, C. W. (1964). The morphology and higher classification of the Coreoidea (Hemiptera‐Heteroptera): Parts I and II. Annals of the Entomological Society of America, 57(6), 670–684. 10.1093/aesa/57.6.670.

[ece311328-bib-0041] Schaefer, C. W. (1993). The Pentatomomorpha (Hemiptera: Heteroptera): An annotated outline of its systematics history. European Journal of Entomology, 90(2), 105–122. https://www.eje.cz/artkey/eje‐199302‐0001.php

[ece311328-bib-0043] Schuh, R. T. (1986). The influence of cladistics on heteropteran classification. Annual Review of Entomology, 31, 67–93. 10.1146/annurev.en.31.010186.000435

[ece311328-bib-0044] Schuh, R. T. , & Slater, J. A. (1995). True bugs of the world (Hemiptera: Heteroptera): Classification and natural history. Entomologische Berichten (Amsterdam), 81(2), 77. https://www.biodiversitylibrary.org/part/180775

[ece311328-bib-0045] Silvestro, D. , & Michalak, I. (2012). RaxmlGUI: A graphical front‐end for RAxML. Organisms, Diversity and Evolution, 12(4), 335–337. https://link.springer.com/article/10.1007/s13127‐011‐0056‐0

[ece311328-bib-0046] Song, F. , Li, H. , Jiang, P. , Zhou, X. , Liu, J. , Sun, C. , Vogler, A. P. , & Cai, W. (2016). Capturing the phylogeny of holometabola with mitochondrial genome data and Bayesian site‐heterogeneous mixture models. Genome Biology and Evolution, 8(5), 1411–1426. 10.1093/gbe/evw086 27189999 PMC4898802

[ece311328-bib-0047] Štys, P. (1961). Morphology of the abdomen and female ectodermal genitalia of the trichophorous Heteroptera and bearing on their classification. Verhandlungen XI International Entomology (Kongress), 1, 37–43.

[ece311328-bib-0048] Štys, P. (1967). Monograph of Malcinae, with reconsideration of morphology and phylogeny of related groups (Heteroptera, Malcidae). Acta Entomologica Musei Nationalis Pragae, 37, 351–516. https://dialnet.unirioja.es/descarga/articulo/6441773.pdf

[ece311328-bib-0049] Štys, P. , & Kerzhner, I. (1975). The rank and nomenclature of higher taxa in recent Heteroptera. Acta Entomologica Bohemoslovaca, 72(2), 65–79. https://pascal‐francis.inist.fr/vibad/index.php?action=getRecordDetail&idt=PASCAL7536011074

[ece311328-bib-0050] Talavera, G. , & Castresana, J. (2007). Improvement of phylogenies after removing divergent and ambiguously aligned blocks from protein sequence alignments. Systematic Biology, 56(4), 564–577. 10.1080/10635150701472164 17654362

[ece311328-bib-0051] Tian, X. X. , Xie, Q. , Li, M. , Gao, C. Q. , Cui, Y. , Xi, L. , & Bu, W. J. (2011). Phylogeny of pentatomomorphan bugs (Hemiptera‐Heteroptera: Pentatomomorpha) based on six hox gene fragments. Zootaxa, 2888(2888), 57–68. 10.11646/zootaxa.2888.1.5

[ece311328-bib-0052] Timmermans, M. J. , Barton, C. , Haran, J. , Ahrens, D. , Culverwell, C. L. , Ollikainen, A. , Dodsworth, S. , Foster, P. G. , Bocak, L. , & Vogler, A. P. (2015). Family‐level sampling of mitochondrial genomes in coleoptera: Compositional heterogeneity and phylogenetics. Genome Biology and Evolution, 8(1), 161–175. 10.1093/gbe/evv241 26645679 PMC4758238

[ece311328-bib-0053] Wang, Y. H. , Cui, Y. , Rédei, D. , Baňař, P. , Xie, Q. , Štys, P. , Damgaard, J. , Chen, P. P. , Yi, W. B. , Wang, Y. , Dang, K. , Li, C. R. , & Bu, W. J. (2016). Phylogenetic divergences of the true bugs (Insecta: Hemiptera: Heteroptera), with emphasis on the aquatic lineages: The last piece of the aquatic insect jigsaw originated in the late Permian/early Triassic. Cladistics, 32(4), 404–407. 10.1111/cla.12137 34740299

[ece311328-bib-0054] Wang, Y. H. , Wu, H. Y. , Redei, D. , Xie, Q. , Chen, Y. , Chen, P. P. , Dong, Z. E. , Dang, K. , Damgaard, J. , Stys, P. , Wu, Y. Z. , Luo, J. Y. , Sun, X. Y. , Hartung, V. , Kuechler, S. M. , Liu, Y. , Liu, H. X. , & Bu, W. J. (2019). When did the ancestor of true bugs become stinky? Disentangling the phylogenomics of Hemiptera‐Heteroptera. Cladistics, 35(1), 42–66. 10.1111/cla.12232 34636080

[ece311328-bib-0055] Weirauch, C. , & Schuh, R. T. (2011). Systematics and evolution of Heteroptera: 25 years of progress. Annual Review of Entomology, 56(1), 487–510. 10.1146/annurev-ento-120709-144833 20822450

[ece311328-bib-0056] Weirauch, C. , Schuh, R. T. , Cassis, G. , & Wheeler, W. C. (2019). Revisiting habitat and lifestyle transitions in Heteroptera (Insecta: Hemiptera): Insights from a combined morphological and molecular phylogeny. Cladistics, 35, 67–105. 10.1111/cla.12233 34622978

[ece311328-bib-0057] Wortley, A. H. , Rudall, P. J. , Harris, D. J. , & Scotland, R. W. (2005). How much data are needed to resolve a difficult phylogeny? Case study in Lamiales. Systematic Biology, 54(5), 697–709. 10.1080/10635150500221028 16195214

[ece311328-bib-0058] Wu, Y. Z. , Yu, S. S. , Wang, Y. H. , Wu, H. Y. , Li, X. R. , Men, X. Y. , Zhang, Y. W. , Rédei, D. , Xie, Q. , & Bu, W. J. (2016). The evolutionary position of Lestoniidae revealed by molecular autapomorphies in the secondary structure of rRNA besides phylogenetic reconstruction (Insecta: Hemiptera: Heteroptera). Zoological Journal of the Linnean Society, 177(4), 750–763. 10.1111/zoj.12385

[ece311328-bib-0059] Xie, Q. , Bu, W. J. , & Zheng, L. Y. (2005). The Bayesian phylogenetic analysis of the 18S rRNA sequences from the main lineages of Trichophora (Insecta: Heteroptera: Pentatomomorpha). Molecular Phylogenetics and Evolution, 34(2), 448–451. 10.1016/j.ympev.2004.10.015 15619455

[ece311328-bib-0060] Xie, Q. , Tian, X. , Qin, Y. , & Bu, W. (2009). Phylogenetic comparison of local length plasticity of the small subunit of nuclear rDNAs among all Hexapoda orders and the impact of hyper‐length‐variation on alignment. Molecular Phylogenetics and Evolution, 50(2), 310–316. 10.1016/j.ympev.2008.10.025 19027081

[ece311328-bib-0061] Xie, Y. , Wu, G. , Tang, J. , Luo, R. , Patterson, J. , Liu, S. , Huang, W. , He, G. , Gu, S. , Li, S. , Zhou, X. , Lam, T. W. , Li, Y. , Xu, X. , Wong, G. K. , & Wang, J. (2014). SOAPdenovo‐trans: de novo transcriptome assembly with short RNA‐seq reads. Bioinformatics, 30(12), 1660–1666. 10.1093/bioinformatics/btu077 24532719

[ece311328-bib-0062] Yang, H. , Li, T. , Dang, K. , & Bu, W. (2018). Compositional and mutational rate heterogeneity in mitochondrial genomes and its effect on the phylogenetic inferences of Cimicomorpha (Hemiptera: Heteroptera). BMC Genomics, 19(1), 264. https://bmcgenomics.biomedcentral.com/articles/10.1186/s12864‐018‐4650‐9 29669515 10.1186/s12864-018-4650-9 PMC5907366

[ece311328-bib-0063] Ye, F. , Kment, P. , Rédei, D. , Luo, J. Y. , Wang, Y. H. , Kuechler, S. M. , Zhang, W. W. , Chen, P. P. , Wu, H. Y. , Wu, Y. Z. , Sun, X. Y. , Ding, L. , Wang, Y. R. , & Xie, Q. (2022). Diversification of the phytophagous lineages of true bugs (Insecta: Hemiptera: Heteroptera) shortly after that of the flowering plants. Cladistics, 38(4), 403–428. 10.1111/cla.12501 35349192

[ece311328-bib-0064] Yu, S. , Wang, Y. , Redei, D. , Xie, Q. , & Bu, W. (2013). Secondary structure models of 18S and 28S rRNAs of the true bugs based on complete rDNA sequences of Eurydema maracandica Oshanin, 1871 (Heteroptera, Pentatomidae). ZooKeys, 319(319), 363–377. 10.3897/zookeys.319.4178 PMC3764533 24039531

[ece311328-bib-0065] Yuan, M. L. , Zhang, Q. L. , Guo, Z. L. , Wan, J. , & Shen, Y. Y. (2015). Comparative mitogenomic analysis of the superfamily Pentatomoidea (Insecta: Hemiptera: Heteroptera) and phylogenetic implications. BMC Genomics, 16(1), 460. 10.1186/s12864-015-1679-x 26076960 PMC4469028

[ece311328-bib-0066] Zhang, D. L. , Gao, J. H. , Li, M. , Yuan, J. J. , Liang, J. Y. , Yang, H. H. , & Bu, W. J. (2019). The complete mitochondrial genome of *Tetraphleps aterrimus* (Hemiptera: Anthocoridae): Genomic comparisons and phylogenetic analysis of Cimicomorpha. International Journal of Biological Macromolecules, 130, 369–377. 10.1016/j.ijbiomac.2019.02.130 30802516

[ece311328-bib-0067] Zhang, D. L. , Li, M. , Li, T. , Yuan, J. J. , & Bu, W. J. (2018). A mitochondrial genome of Micronectidae and implications for its phylogenetic position. International Journal of Biological Macromolecules, 119, 747–757. 10.1016/j.ijbiomac.2018.07.191 30075212

[ece311328-bib-0068] Zhao, Q. , Wang, J. , Wang, M. , Cai, B. , Zhang, H. F. , & Wei, J. F. (2018). Complete mitochondrial genome of *Dinorhynchus dybowskyi* (Hemiptera: Pentatomidae: Asopinae) and phylogenetic analysis of Pentatomomorpha species. Journal of Insect Science, 18(2), 1–12. 10.1093/jisesa/iey031 PMC5905379 29718506

[ece311328-bib-0069] Zhao, W. Q. , Liu, D. J. , Jia, Q. , & Zhang, H. F. (2021). Characterization of the complete mitochondrial genome of *Myrmus lateralis* (Heteroptera, Rhopalidae) and its implication for phylogenetic analyses. ZooKeys, 1070, 13–30. 10.3897/zookeys.1070.72742 34819768 PMC8599289

[ece311328-bib-0070] Zhao, W. Q. , Zhao, Q. , Li, M. , Wei, J. F. , Zhang, X. H. , & Zhang, H. F. (2019). Comparative mitogenomic analysis of the *Eurydema* genus in the context of representative Pentatomidae (Hemiptera: Heteroptera) taxa. Journal of Insect Science, 19(6), 1–12. 10.1093/jisesa/iez122 PMC6913905 31841604

[ece311328-bib-0071] Zhou, J. , Liu, X. , Stones, D. S. , Xie, Q. , & Wang, G. (2011). MrBayes on a graphics processing unit. Bioinformatics, 27(9), 1255–1261. 10.1093/bioinformatics/btr140 21414986

