# Supplementary material for: Phylogenetic placement and comparative analysis of the mitochondrial genomes of Idiostoloidea (Hemiptera: Heteroptera)
Source: Ecol Evol. 2024 May 2;14(5):e11328. doi: 10.1002/ece3.11328 (PMC11063732; doi:10.1002/ece3.11328)

**Phylogenetic placement and comparative analysis of the mitochondrial genomes of Idiostoloidea (Hemiptera: Heteroptera)**

Danli Zhang^1^, XiaoYan Chen^1^, Jingjing Yang^1^, Wenbo Yi^2^, Qiang Xie^2^, HuanHuan Yang^3^, Merrill H. Sweet^4^, Wenjun Bu^2,*^, Teng Li^2,5,*^

^1^College of Biological Sciences and Technology, Taiyuan Normal University, Jinzhong, China

^2^Institute of Entomology, College of Life Sciences, Nankai University, Tianjin, China

^3^School of Bioengineering, Qilu University of Technology (Shandong Academy of Sciences), Jinan, China

^4^Department of Entomology, Plant Pathology and Weed Science, New Mexico State University, Las Cruces, USA

^5^School of Biological Sciences, University of Auckland, Auckland, New Zealand

*Corresponding author

Email addresses:

DZ: [danlizhang2013@163.com](mailto:danlizhang2013@126.com)

XC: xy15935990119@163.com

JY: 3111690835@qq.com

WY: yiwb1988@yeah.net

QX: qiangxie@nankai.edu.cn

HY: yanghuanhuan1116@163.com

MHS: [msweet@bio.tamu.edu](mailto:msweet@bio.tamu.edu)

WB: [wenjunbu@nankai.edu.cn](mailto:wenjunbu@nankai.edu.cn)

TL: teng.li@auckland.ac.nz

Tel: 0086-22-23498957, Fax: 0086-22-23498957.

## Supplementary Table S1. Summary of sample information and GenBank accession numbers used in this study.

| **Infraorder (bold)**  **Superfamily (not bold)** | **Family** | **Species** | **Accession number of mt-genome** | **Species** | **Accession number of 18SrDNA** | **Species** | **Accession number of 28SrDNA** |
| --- | --- | --- | --- | --- | --- | --- | --- |
| **Leptopodomorpha** |  |  |  |  |  |  |  |
| Saldoidea | Saldidae | *Saldula arsenjevi* | NC_012463 | *Saldula saltatoria* | KJ461170 | *Saldula saltatoria* | KJ461213 |
| **Cimicomorpha** |  |  |  |  |  |  |  |
| Cimicoidea | Anthocoridae | *Orius niger* | NC_012429 | *Anthocoris pilosus* | KJ461226 | *Anthocoris pilosus* | KJ461268 |
| Reduvioidea | Reduviidae | *Triatoma dimidiate* | NC_002609 | *Triatoma dimidiate* | AJ243328 | *Coranus spiniscutis* | KJ461229 |
| **Pentatomomorpha** |  |  |  |  |  |  |  |
| Aradoidea | Aradidae | *Neuroctenus parus* | NC_012459 | ***/*** | ***/*** | ***/*** | ***/*** |
|  |  | *Brachyrhynchus hsiaoi* | NC_022670 | *Mezira* sp. | KJ461285 | *Mezira* sp. | KJ461189 |
| Pentatomoidea | Pentatomidae | *Eurydema gebleri* | NC_027489 | *Eurydema maracandica* | JX997807 | *Eurydema maracandica* | JX997806 |
|  |  | *Dolycoris baccarum* | NC_020373 | *Dolycoris penicillatus* | KJ535863 | *Dolycoris penicillatus* | KJ535863 |
|  | Tessaratomidae | *Eusthenes cupreus* | NC_022449 | *Eusthenes cupreus* | KJ535869 | *Eusthenes cupreus* | KJ535869 |
|  | Urostylididae | *Urochela quadrinotata* | NC_020144 | *Urochela luteovaria* | KJ461205 | *Urochela luteovaria* | KJ461306 |
|  | Cydnidae | *Macroscytus gibbulus* | NC_012457 | *Macroscytus fraterculus* | KJ535876 | *Macroscytus fraterculus* | KJ535876 |
|  | Plataspidae | *Coptosoma bifaria* | NC_012449 | *Coptosoma bifarium* | KJ461259 | *Coptosoma bifarium* | KJ461239 |
| Idiostoloidea | Idiostolidae | *Monteithocoris hirsutus* | OR134606* | *Monteithocoris hirsutus* | OR223290* | *Monteithocoris hirsutus* | OR223290* |
|  | Henicocoridae | *Henicocoris* sp. | OR189406* | *Henicocoris* sp. | OR223291* | *Henicocoris* sp. | OR223291* |
| Lygaeoidea | Berytidae | *Yemmalysus parallelus* | NC_012464 | *Neoneides muticus* | AY252412 | *Neoneides muticus* | AY252631 |
|  | Colobathristidae | *Phaenacantha marcida* | NC_012460 | ***/*** | ***/*** | ***/*** | ***/*** |
|  | Heterogastridae | *Heterogaster chinensis* | OR134605* | *Heterogaster cathariae* | KJ461278 | *Heterogaster cathariae* | KJ461302 |
|  | Malcidae | *Malcus inconspicuous* | NC_012458 | ***/*** | ***/*** | ***/*** | ***/*** |
|  |  | *Chauliops fallax* | JX839706 | ***/*** | ***/*** | ***/*** | ***/*** |
|  | Oxycarenidae | *Oxycarenus pallens* | OR134607* | ***/*** | ***/*** | ***/*** | ***/*** |
|  | Pachygronthidae | *Pachygrontha antennata* | OR134608* | *Pachygrontha antennata* | KJ461210 | *Pachygrontha antennata* | KJ461207 |
|  | Piesmatidae | *Parapiesma salsolae* | OR134773* | *Piesma maculatum* | KJ461171 | *Piesma maculatum* | KJ461169 |
|  | Rhyparochromidae | *Panaorus albomaculatus* | KX216853 | ***/*** | ***/*** | ***/*** | ***/*** |
|  | Geocoridae | *Geocoris papRennis* | NC_012424 | *Geocoris ater* | KJ461218 | *Geocoris ater* | KJ461309 |
| Pyrrhocoroidea | Largidae | *Physopelta gutta* | NC_012432 | *Physopelta gutta* | KJ461164 | *Physopelta gutta* | KJ461255 |
|  | Pyrrhocoridae | *Dysdercus cingulatus* | NC_012421 | *Dysdercus cingulatus* | KJ461263 | *Dysdercus cingulatus* | KJ461235 |
| Coreoidea | Alydidae | *Riptortus pedestris* | NC_012462 | *Riptortus pedestris* | AB725684 | *Riptortus pedestris* | AB725684 |
|  | Coreidae | *Hydaropsis longirostris* | NC_012456 | *Cletus punctiger* | KJ461173 | *Cletus punctiger* | KJ461219 |
|  | Rhopalidae | *Stictopleurus subviridis* | NC_012888 | *Stictopleurus punctatonervosus* | KJ461217 | *Stictopleurus punctatonervosus* | KJ461286 |

^*^Represents the new sequences in this study.

## Supplementary Table S2. Collecting information of six newly sequenced species in the present study.

| **Superfamily (bold) Family (not bold)** | **Species** | **Collecting locality** | **Date** |
| --- | --- | --- | --- |
| **Idiostoloidea** |  |  |  |
| Idiostolidae | *Monteithocoris hirsutus* | Liffey Forest Reserve, Tasmania, Australia | 7 March 1998 |
| Henicocoridae | *Henicocoris* sp. | Great Otway National Park, Victoria, Australia | 15 March 1998 |
| **Lygaeoidea** |  |  |  |
| Heterogastridae | *Heterogaster chinensis* | Shayuan (29°47'N, 109°49'E), Hefeng County,Hubei Province, China | 17 July 1999 |
| Oxycarenidae | *Oxycarenus pallens* | Nankai University (39°06’N, 117°10’E), Tianjin, China | 20 May 2002 |
| Pachygronthidae | *Pachygrontha antennata* | Jiugong Mountain (29°22'N, 114°34'E), Tongshan County, Hubei Province, China | 2 August 2010 |
| Piesmatidae | *Parapiesma salsolae* | Cangyan Mountain (37°50'N, 114°10'E), Jingxing County, Hebei Province, China | 18 July 2001 |

## Supplementary Table S3. Primers designed for mt-genome amplification of four newly sequenced Lygaeoidea sepcies in this study.

| **Primer^a^** | **Sequence (5’-3’)** | **Region** |
| --- | --- | --- |
| COIF | GGAACAGGATGAACAGTTTACCCTCC | COI |
| COIR | TCTGAATATCGTCGAGGTATTCC |  |
|  |  |  |
| CytBF | TATGTTCTTCCCTGAGGACAAATATC | CytB |
| CytBR | ATGAACTGGTGTTACTAATGGGTTTGCTGG |  |
|  |  |  |
| Het1F | CAGTTACCAGGATTTGGACTTATTTCAC | COI-CytB (9kb fragment) |
| Het1R | TGATCTTCTTGTTTGGCTGAGACTAATC |  |
|  |  |  |
| Het2F | AGCCTTCATCGTAATAGGCATAGTGTT | CytB-COI (7kb fragment) |
| Het2R | TATGTGTGAAATAAGTCCAAATCCTG |  |
|  |  |  |
| Oxy1F | TAGCAGGAGTATCATCAATTTTAGGAGC | COI-CytB (9kb fragment) |
| Oxy1R | GTAGAAAATGTAAAGTAAAGAATCGTGT |  |
|  |  |  |
| Oxy2F | CAAACAGGAAGTAATAACCCTCTAGGATT | CytB-COI (7kb fragment) |
| Oxy2R | AATGAAGTATTAAAGTTACGGTCTGT |  |
|  |  |  |
| Pac1F | CATTTAGCAGGAGTATCATCAATTTTAGGAGC | COI-CytB (9kb fragment) |
| Pac1R | AATAGGAAATATCATTCAGGTTGAATATG |  |
|  |  |  |
| Pac2F | TATGAGGGGGATTCTCAGTTGATAATGC | CytB-COI (7kb fragment) |
| Pac2R | TCGTCCAAAGAATCAAAATAAGTGCTG |  |
|  |  |  |
| Pie1F | GTACCTGTATTAGCGGGAGCAATCACT | COI-CytB (9kb fragment) |
| Pie1R | TTGGGAATGGAGCGTAAAATAGAATAGGC |  |
|  |  |  |
| Pie2F | GATCGCCTATTCTATTTTACGCTCCAT | CytB-COI (7kb fragment) |
| Pie2R | ACTGCTCCCGCTAATACAGGTAAAGATA |  |

^a^ The primers of COIF, COIR, CytBF, and CytBR were modified from Hua et al. (2008). Each pair of primers special to each species to rate two long fragments of mt-genome were named with the first three letters of their family name.

## Supplementary Table S4. The best partitioning strategies detected by PartitionFinder for different data matrices.

| **Data matrices** | **Best model** | **Number of partitions** | **Partition scheme** |
| --- | --- | --- | --- |
| PCG12 | Bayesian, GTR+I+G  ML, GTRGAMMAI | 5 | COI 1st + CytB 1st + COIII 1st + COII 1st; COIII 2nd + ATP6 2nd + COI 2nd + CytB 2nd + COII 2nd; ND2 1st + ND3 1st + ATP8 1st + ND6 1st + ATP6 1st + ATP8 2nd; ND1 1st + ND5 1st + ND4L 1st + ND4 1st; ND3 2nd + ND2 2nd + ND6 2nd + ND4L 2nd + ND4 2nd + ND5 2nd + ND1 2nd |
|  |  |  |  |
| PCG12rDNA | Bayesian, GTR+I+G  ML, GTRGAMMAI | 6 | COI 1st + CytB 1st + COIII 1st + COII 1st; COIII 2nd + ATP6 2nd + COI 2nd + CytB 2nd + COII 2nd; ND2 1st + ND3 1st + ATP8 1st + ND6 1st + ATP6 1st + ATP8 2nd; ND1 1st + ND5 1st + ND4L 1st + ND4 1st; ND3 2nd + ND2 2nd + ND6 2nd + ND4L 2nd + ND4 2nd + ND5 2nd + ND1 2nd; 18S + 28S rDNA |
|  |  |  |  |
| PCG12RNArDNA | Bayesian, GTR+I+G  ML, GTRGAMMAI | 7 | ND3 1st + ATP8 1st + ND6 1st + ATP6 1st + ATP8 2nd + COI 1st + CytB 1st + COII 1st + COIII 1st; COIII 2nd + ATP6 2nd + COI 2nd + CytB 2nd + COII 2nd; ND1 1st + ND5 1st + ND4 1st + ND4L 1st; ND3 2nd + ND2 2nd + ND6 2nd + ND4L 2nd + ND4 2nd + ND5 2nd + ND1 2nd; ND2 1st + 12S; 18S + 28S rDNA; 16S |

## Supplementary Table S5. Fossils used in this study for calibrations and divergence time estimation.

| **Calibration nodes** | **Fossils** | **Minimum age constraint for group (Ma)** | **Soft maximum bound (97.5% probability)** | **Reference** |
| --- | --- | --- | --- | --- |
| **Pentatomomorpha** | *Protocoris indistinctus* (Protocoridae) | **201.3** | **230** | Popov et al. 1994 |
| **Aradoidea** | *Aradus nicholasi* (Aradidae) | **125** | **170** | Popov 1989  Song et al. 2016 |
| **Pentatomoidea** | *Cretacoris scutellinus* (Cydnidae) | **132.9** | **180** | Popov 1990 |
| **Coreoidea** | *Originicorizus pyriformis* (Rhopalidae) | **166.1** | **200** | Yao et al. 2006 |
| **Pyrrhocoroidea** | *Mesopyrrhocoris fasciata* (Pyrrhocoridae) | **125** | **170** | Hong and Wang 1990 |
| **Lygaeoidea** | *Leipolygaeus similis* (Lygaeidae) | 170.3 | **200** | Lin 1985 Shcherbakov 2007 |

Supplementary Table S6. Organization of the mitochondrial genome of *Monteithocoris hirsutus.*

| Gene | Strand | Position | Anticodon | Size(bp) | Start  codon | Stop  codon | Intergenic  Nucleotides* |
| --- | --- | --- | --- | --- | --- | --- | --- |
| tRNA-Ile | J | 1-67 | GAT | 67 |  |  |  |
| tRNA-Gln | N | 73-141 | TTG | 69 |  |  | 5 |
| tRNA-Met | J | 153-219 | CAT | 67 |  |  | 11 |
| ND2 | J | 221-1228 |  | 1008 | ATG | TAA | 1 |
| tRNA-Trp | J | 1248-1317 | TCA | 70 |  |  | 19 |
| tRNA-Cys | N | 1310-1378 | GCA | 69 |  |  | -8 |
| tRNA-Tyr | N | 1390-1453 | GTA | 64 |  |  | 11 |
| COI | J | 1457-2995 |  | 1539 | TTG | TAA | 3 |
| tRNA-Leu | J | 2991-3055 | TAA | 65 |  |  | -5 |
| COII | J | 3056-3736 |  | 681 | TTG | TAA | 0 |
| tRNA-Lys | J | 3744-3817 | CTT | 74 |  |  | 7 |
| tRNA-Asp | J | 3847-3913 | GTC | 67 |  |  | 29 |
| ATPase8 | J | 3914-4078 |  | 165 | ATA | TAA | 0 |
| ATPase6 | J | 4072-4740 |  | 669 | ATG | TAA | -7 |
| COIII | J | 4748-5536 |  | 789 | ATG | TAA | 7 |
| tRNA-Gly | J | 5548-5609 | TCC | 62 |  |  | 11 |
| ND3 | J | 5610-5961 |  | 352 | ATC | T- | 0 |
| tRNA-Ala | J | 5962-6022 | TGC | 61 |  |  | 0 |
| tRNA-Arg | J | 6025-6088 | TCG | 64 |  |  | 1 |
| tRNA-Asn | J | 6091-6154 | GTT | 64 |  |  | 3 |
| tRNA-Ser | J | 6154-6222 | GCT | 69 |  |  | -1 |
| tRNA-Glu | J | 6224-6287 | TTC | 64 |  |  | 1 |
| tRNA-Phe | N | 6294-6359 | GAA | 66 |  |  | 6 |
| ND5 | N | 6359-8068 |  | 1710 | ATG | TAA | -1 |
| tRNA-His | N | 8071-8134 | GTG | 64 |  |  | 2 |
| ND4 | N | 8144-9466 |  | 1323 | ATG | TAA | 9 |
| ND4L | N | 9460-9756 |  | 297 | ATG | TAA | -7 |
| tRNA-Thr | J | 9760-9824 | TGT | 65 |  |  | 3 |
| tRNA-Pro | N | 9825-9892 | TGG | 68 |  |  | 0 |
| ND6 | J | 9901-10392 |  | 492 | ATA | TAA | 8 |
| Cytb | J | 10393-11532 |  | 1140 | ATA | TAA | 0 |
| tRNA-Ser | J | 11532-11599 | TGA | 68 |  |  | -1 |
| ND1 | N | 11621-12544 |  | 924 | ATG | TAA | 21 |
| tRNA-Leu | N | 12545-12609 | TAG | 65 |  |  | 0 |
| 16S rRNA | N | 12610-13875 |  | 1266 |  |  | 0 |
| tRNA-Val | N | 13876-13943 | TAC | 68 |  |  | 0 |
| 12S rRNA | N | 13944-14739 |  | 796 |  |  | 0 |
| Control region |  | 14740-16632 |  | 1893 |  |  | 0 |

*Numbers correspond to nucleotides separating a gene from an upstream one; negative numbers indicate that adjacent genes overlap.

Supplementary Table S7. Organization of the mitochondrial genome of *Henicocoris* sp.

| Gene | Strand | Position | Anticodon | Size(bp) | Start  codon | Stop  codon | Intergenic  Nucleotides* |
| --- | --- | --- | --- | --- | --- | --- | --- |
| tRNA-Ile | J | 1-65 | GAT | 65 |  |  |  |
| tRNA-Gln | N | 69-137 | TTG | 69 |  |  | 3 |
| tRNA-Met | J | 143-209 | CAT | 67 |  |  | 5 |
| ND2 | J | 210-1211 |  | 1002 | ATA | TAA | 0 |
| tRNA-Trp | J | 1211-1275 | TCA | 65 |  |  | -1 |
| tRNA-Cys | N | 1268-1335 | GCA | 68 |  |  | -8 |
| tRNA-Tyr | N | 1336-1402 | GTA | 67 |  |  | 0 |
| COI | J | 1406-2939 |  | 1534 | TTG | T- | 3 |
| tRNA-Leu | J | 2940-3004 | TAA | 65 |  |  | 0 |
| COII | J | 3005-3685 |  | 681 | TTG | TAA | 0 |
| tRNA-Lys | J | 3686-3752 | CTT | 67 |  |  | 0 |
| tRNA-Asp | J | 3769-3832 | GTC | 64 |  |  | 16 |
| ATPase8 | J | 3833-3997 |  | 165 | ATC | TAA | 0 |
| ATPase6 | J | 3991-4656 |  | 666 | ATG | TAA | -7 |
| COIII | J | 4658-5446 |  | 789 | ATG | TAA | 1 |
| tRNA-Gly | J | 5461-5523 | TCC | 63 |  |  | 14 |
| ND3 | J | 5524-5889 |  | 366 | ATA | TAA | 0 |
| tRNA-Ala | J | 5876-5936 | TGC | 61 |  |  | -14 |
| tRNA-Arg | J | 5938-6000 | TCG | 63 |  |  | 1 |
| tRNA-Asn | J | 6006-6073 | GTT | 68 |  |  | 5 |
| tRNA-Ser | J | 6073-6141 | GCT | 69 |  |  | -1 |
| tRNA-Glu | J | 6141-6207 | TTC | 67 |  |  | -1 |
| tRNA-Phe | N | 6206-6269 | GAA | 64 |  |  | -2 |
| ND5 | N | 6272-7984 |  | 1713 | ATG | TAA | 2 |
| tRNA-His | N | 7986-8053 | GTG | 68 |  |  | 1 |
| ND4 | N | 8059-9381 |  | 1323 | ATG | TAA | 5 |
| ND4L | N | 9375-9662 |  | 288 | GTG | TAA | -7 |
| tRNA-Thr | J | 9666-9729 | TGT | 64 |  |  | 3 |
| tRNA-Pro | N | 9730-9794 | TGG | 65 |  |  | 0 |
| ND6 | J | 9797-10294 |  | 498 | ATA | TAA | 2 |
| Cytb | J | 10295-11434 |  | 1140 | ATG | TAA | 0 |
| tRNA-Ser | J | 11434-11502 | TGA | 69 |  |  | -1 |
| ND1 | N | 11524-12450 |  | 927 | ATT | TAA | 21 |
| tRNA-Leu | N | 12451-12517 | TAG | 67 |  |  | 0 |
| 16S rRNA | N | 12518-13779 |  | 1262 |  |  | 0 |
| tRNA-Val | N | 13780-13847 | TAC | 68 |  |  | 0 |
| 12S rRNA | N | 13848-14639 |  | 792 |  |  | 0 |
| Control region |  | 14640-16013 |  | 1374 |  |  | 0 |

*Numbers correspond to nucleotides separating a gene from an upstream one; negative numbers indicate that adjacent genes overlap.

## Supplementary Figure S1. AliGROOVE analysis for each codon positions of protein-coding genes (PCGs) and three datasets, respectively.


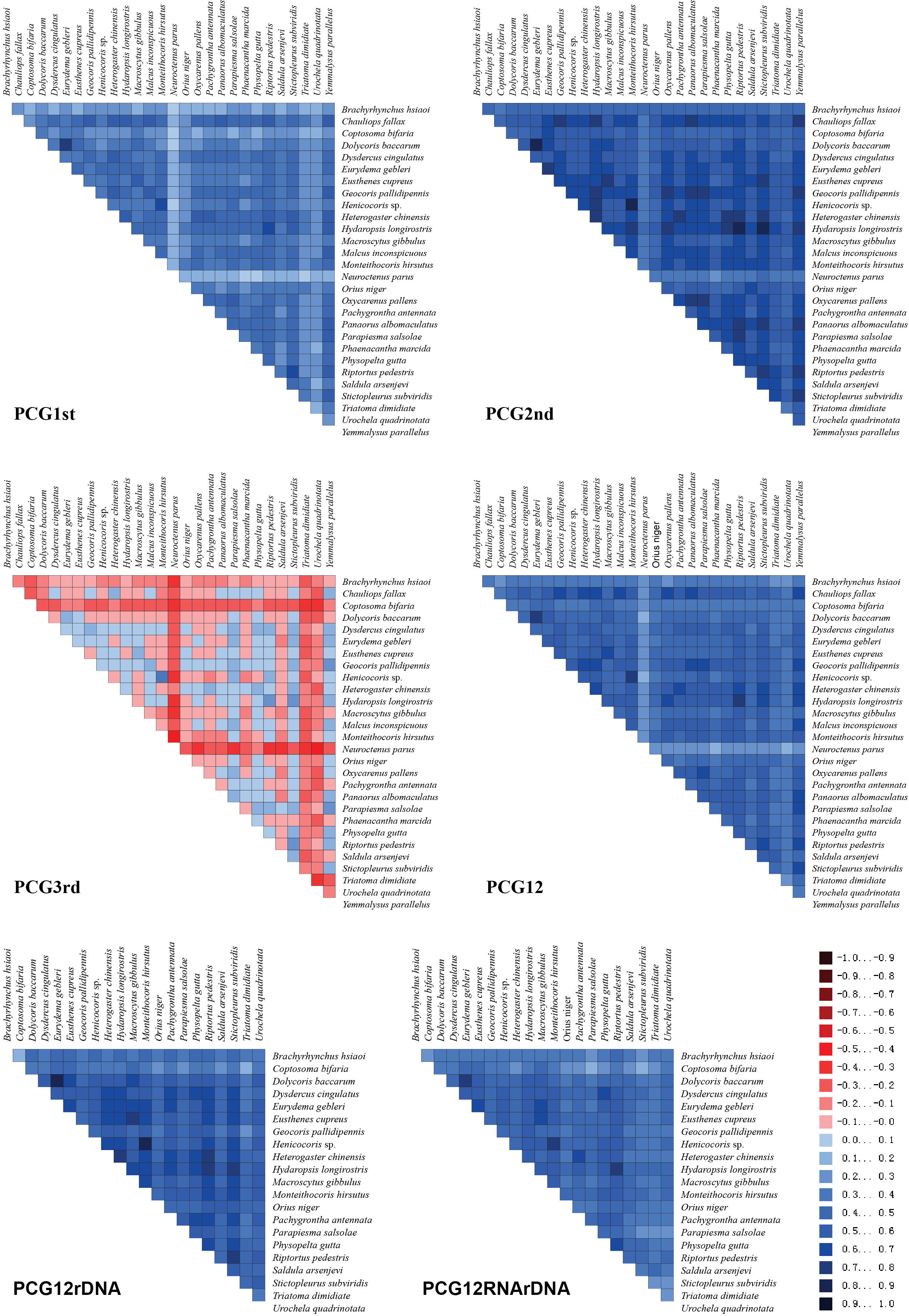
PCG1st, PCG2nd and PCG3rd indicate the first, second and third codon position of PCGs, respectively. The coloured squares show the AliGROOVE similarity scores with the blue (positive scores) indicate the level of non-randomized accordancy, whereas the read (negative scores) indicate the level of heterogeneity between pairwise sequence comparisons.

Supplementary Figure S2. Secondary structures of the 22 transfer RNA genes of *Monteithocoris hirsutus*. Dashes (−) indicate Watson-Crick pairing. GU bonds by asterisk, and the other non-Watson-Crick interactions are illustrated by hollow circles.

**
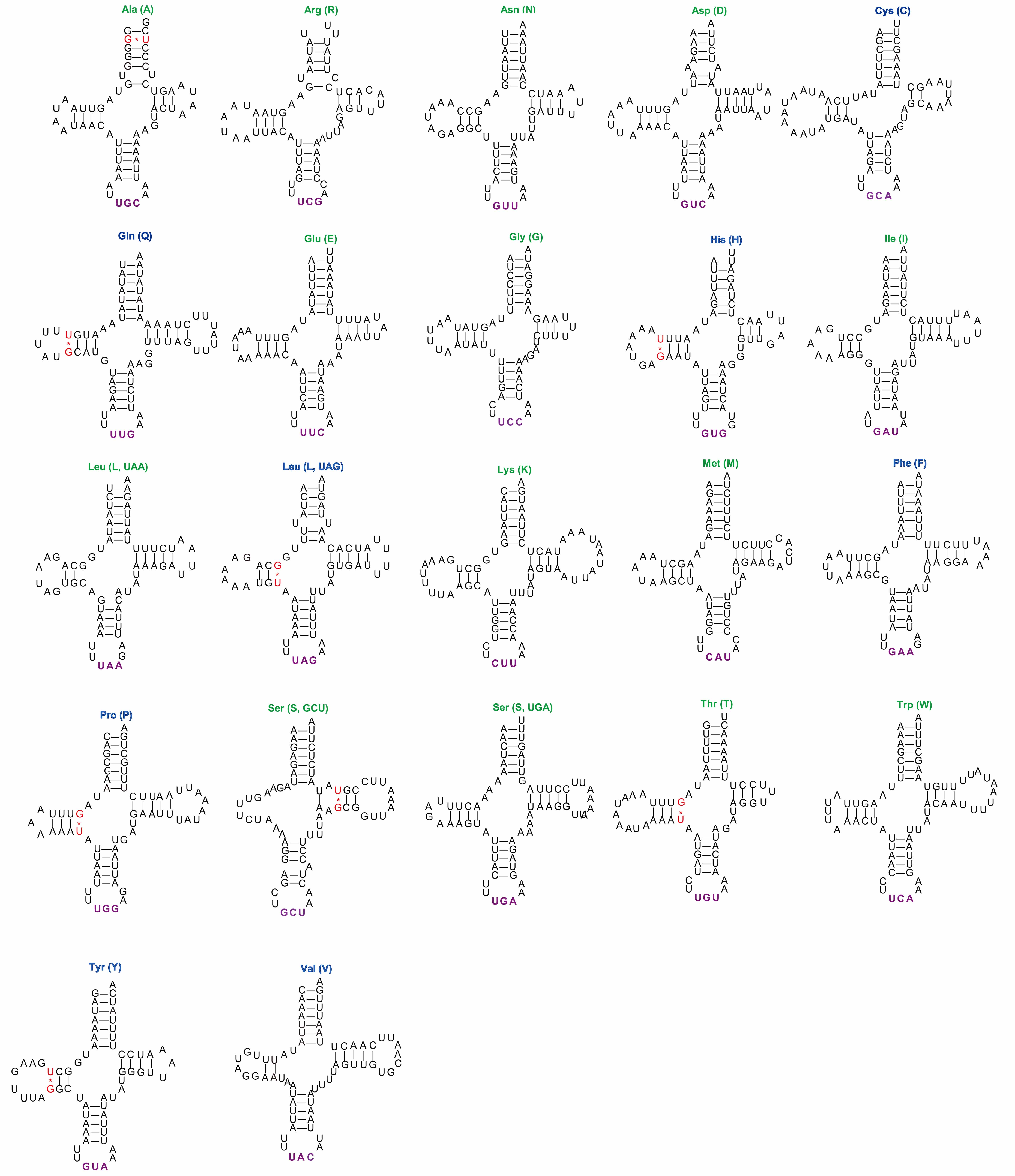
**

Supplementary Figure S3. Secondary structures of the 22 transfer RNA genes of *Henicocoris* sp. Dashes (−) indicate Watson-Crick pairing. GU bonds by asterisk, and the other non-Watson-Crick interactions are illustrated by hollow circles.


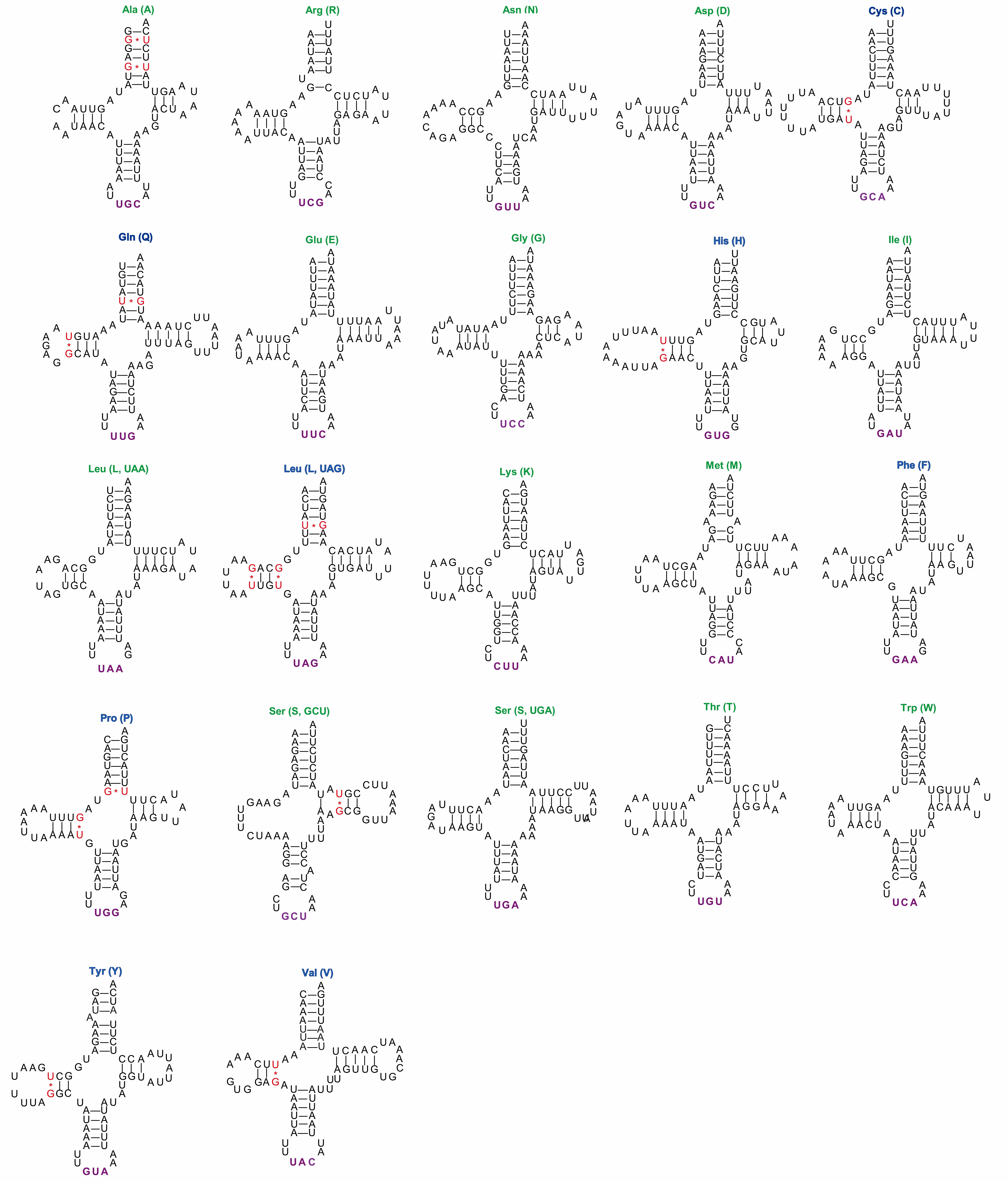

Supplement: Supplementary file 1 — Appendix S1. [file ECE3-14-e11328-s001.docx]
